# A New Remote Guided Method for Supervised Web-Based Cognitive Testing to Ensure High-Quality Data: Development and Usability Study

**DOI:** 10.2196/28368

**Published:** 2022-01-06

**Authors:** Victoria Leong, Kausar Raheel, Jia Yi Sim, Kriti Kacker, Vasilis M Karlaftis, Chrysoula Vassiliu, Kastoori Kalaivanan, S H Annabel Chen, Trevor W Robbins, Barbara J Sahakian, Zoe Kourtzi

**Affiliations:** 1 Psychology School of Social Sciences Nanyang Technological University Singapore Singapore; 2 Centre for Research and Development in Learning Nanyang Technological University Singapore Singapore; 3 Lee Kong Chian School of Medicine Nanyang Technological University Singapore Singapore; 4 Department of Psychology University of Cambridge Cambridge United Kingdom; 5 Faculty of Modern and Medieval Languages and Linguistics University of Cambridge Cambridge United Kingdom; 6 National Institute of Education Nanyang Technological University Singapore Singapore; 7 Behavioural and Clinical Neuroscience Institute University of Cambridge Cambridge United Kingdom; 8 Department of Psychiatry University of Cambridge Cambridge United Kingdom

**Keywords:** web-based testing, neurocognitive assessment, COVID-19, executive functions, learning

## Abstract

**Background:**

The global COVID-19 pandemic has triggered a fundamental reexamination of how human psychological research can be conducted safely and robustly in a new era of digital working and physical distancing. Online web-based testing has risen to the forefront as a promising solution for the rapid mass collection of cognitive data without requiring human contact. However, a long-standing debate exists over the data quality and validity of web-based studies. This study examines the opportunities and challenges afforded by the societal shift toward web-based testing and highlights an urgent need to establish a standard data quality assurance framework for online studies.

**Objective:**

This study aims to develop and validate a new supervised online testing methodology, remote guided testing (RGT).

**Methods:**

A total of 85 healthy young adults were tested on 10 cognitive tasks assessing executive functioning (flexibility, memory, and inhibition) and learning. Tasks were administered either face-to-face in the laboratory (n=41) or online using remote guided testing (n=44) and delivered using identical web-based platforms (Cambridge Neuropsychological Test Automated Battery, Inquisit, and i-ABC). Data quality was assessed using detailed trial-level measures (missed trials, outlying and excluded responses, and response times) and overall task performance measures.

**Results:**

The results indicated that, across all data quality and performance measures, RGT data was statistically-equivalent to in-person data collected in the lab (*P*>.40 for all comparisons). Moreover, RGT participants out-performed the lab group on measured verbal intelligence (*P*<.001), which could reflect test environment differences, including possible effects of mask-wearing on communication.

**Conclusions:**

These data suggest that the RGT methodology could help ameliorate concerns regarding online data quality—particularly for studies involving high-risk or rare cohorts—and offer an alternative for collecting high-quality human cognitive data without requiring in-person physical attendance.

## Introduction

### Background

In 2020, the global COVID-19 pandemic brought human lab-based psychological research to an abrupt halt as social distancing measures preventing disease transmission forced the mass closure of laboratory facilities and prevented all but essential human contact, disrupting academic research at a profound level [1,2]. During this period of suspension, the research community has inexorably moved toward remote protocols to replace face-to-face activities. There has been an exponential rise in the use of online platforms such as video conferencing (Zoom [3] and Skype) and online learning [3,4] for day-to-day academic activities, and the use of social media platforms has surged, not just as a means for interacting and connecting with others, but also for participant recruitment and outreach [5]. Concomitantly, interest in online experimental alternatives to in-person cognitive testing has grown significantly [1], and there is an increasing focus on methodological developments that will allow the field to adapt to a changed world where reduced social contact is the new norm [1,6,7]. However, amidst this push toward new online research technologies, core issues of data quality and validity should not be overlooked, and simple assumptions of equivalence between lab-based and online tests (eg, on the basis that the use of similar tasks and platforms is sufficient [8-10]) should not be made. Failure to address these issues could lead to a proliferation of poorly regulated online research studies, worsening the current reproducibility crisis and raising new ethical dilemmas [11]. This study examines the opportunities and challenges afforded by the shift toward web-based testing, highlighting an urgent need to establish a standard data quality assurance framework for online studies and proposing a new supervised online testing methodology, remote guided testing (RGT), which could mitigate some of these challenges and offer an alternative for collecting high-quality human cognitive data within social distancing constraints.

### The Rise of Web-Based Cognitive Testing: Opportunities and Challenges

Cognitive tests are valuable psychological tools used extensively to examine mental executive processes such as learning, decision-making, inhibition, and working memory [12-31]. Tests of executive processes have typically involved pen-and-paper administration in lab-based settings, allowing the experimenter to confirm the participant’s identity, deter dishonesty, and promptly assist with queries or technical problems. The standardized testing environment and equipment further aid to ensure replicability and reproducibility of lab-based protocols [32-37]. However, this traditional in-person approach is time-consuming and highly susceptible to human error [12,32]. Further, since lab-based testing requires participants’ physical attendance in the laboratory, sampling may not be population-representative [38-40]. For instance, Henrich et al [38], Nielsen et al [39], and Arnett [40] report that participants in lab-based studies consist predominantly of Western, educated, industrialized, rich, and democratic populations. An analysis of 6 major American Psychological Association journals [40,41] showed that a significant number of studies reported in these journals relied predominantly on American students. Much of the normative data on psychological and cognitive processes has been obtained from a North American, White, high socioeconomic status, and well-educated demographic, raising the possibility that neuropsychology may be insensitive to cultural and ethnic differences [42,43]. Structural racism, that is, the establishment of a series of dynamics that promote White people as the norm (to the exclusion or minimization of Black and ethnic minority people), may also have led to the routine acceptance of nonrepresentative standardization samples that are primarily White, creating false normative expectations for Black and ethnic minorities [44,45]. These biased practices in psychological assessment have long gone unchallenged, partially due to a prevalent belief in universalism (ie, the theory that cognitive processes are essentially the same across humankind, irrespective of cultural background) [46,47]. This highlights the need for a wider representation of ethnic minority groups and cultures in psychological studies. Web-based testing could help to ameliorate this gap and reach a more diverse global audience for neuropsychological research.

In the current digital age, the mass availability of personal computers and web capability affords new avenues for cognitive testing using more cost-effective, automated, and open approaches [12,48,49]. Accordingly, there is growing momentum in the use of online platforms to assess cognitive function [12,50-52]. Computerized online testing platforms such as Gorilla [50], Inquisit [52], and the Cambridge Neuropsychological Test Automated Battery (CANTAB) [51] can offer several advantages, including (1) simple and precise control of experimental parameters, (2) automatic calculation of key performance indices, (3) access to normative databases, (4) accessibility to a wider population of users (with use of crowdsourcing tools), (5) centralized and secure data storage on professional servers, and (6) relatively low administrative cost per head [32,34,48,53]. Notably, web-based online testing removes the physical constraint of test locations, permitting a much wider (and more representative) demographic reach [34,54]. Further, social media platforms, recruitment portals (eg, Amazon’s MTurk and Prolific Academic), and online advertisements have broadened horizons by increasing ease of participant recruitment and enabling high throughput data collection from large populations, which is less feasible in traditional lab-based settings [49,55-58].

The COVID-19 pandemic has fueled the growth of “telehealth” or remote access to health care services, bringing to the fore particular challenges in providing remote neuropsychological assessments, psychoeducation, and rapport building [59]. Ongoing demand for telehealth services even in the postpandemic era is likely, as these may be valuable solutions when physical presence in the clinic is impossible for other reasons (eg, sickness, workload, etc). Relatedly, remote testing (or tele-testing) is becoming increasingly popular amongst clinicians. Tele-testing, often combined with face-to-face testing, results in a hybrid approach that can cater to the specific needs of patients and their families [59]. Singh and Germane [60] elaborate on this hybrid approach, which they call “hybrid neuropsychology” (HN). HN enables clinicians to effectively and steadily modernize their practice considering individuals' needs and (technological) capabilities and evaluating which tasks are ideal for online or remote administration. Indeed, HN incorporates tele-testing practices and screen-sharing options wherever materials have been digitized properly—bearing similarities to the remote guided methodology proposed in this study. Additionally, rigorous and standardized protocols for web-based test delivery are not yet available for most neuropsychological tasks, limiting the confidence with which clinicians and scientists can adopt these methodologies in daily practice. This study addresses a growing demand for remote methods of neuropsychological measurement in both research and clinical settings by providing one such detailed remote administration protocol for a suite of executive function and cognitive tasks that are highly relevant to clinical assessment.

Further, a long-standing debate still exists regarding the data quality, comparability, replicability, and validity of web-based versus traditional lab-based data collection methods [61,62]. On the one hand, direct comparisons of web-based and lab-based data samples from web pioneers such as Germine and colleagues [63-65] on a series of large-scale web-based studies on memory and perception (testmybrain.org) indicate that the reliability, replicability, and theoretical consistency of self-selected web samples are comparable to lab-collected data in terms of mean performance and performance variability, even with anonymous, uncompensated, and unsupervised participants. On the other hand, concerns have been raised, and not yet fully allayed, about the experimental rigor of web-based testing [36,37], particularly regarding the lack of control over and higher variability of hardware specifications and the test environment [36,37,66]. For instance, a study by Bauer et al [36] reported that most online studies suffer from a lack of environmental control and participant distraction. Further, most online studies do not monitor or report measures of participants’ environment, their equipment specification, or web capability. These data, when reported, typically reveal large variations in the equipment and computer specifications used by participants [34,66]. In a landmark study on computing specifications in online and lab-based studies, Bridges et al [66] compared the pairings of several web-based experimental platforms, such as Gorilla and jsPsych, with different operating systems, such as Windows, macOS, and Ubuntu. In data collected from over 110,000 trials, macOS yielded the worst performance across all experimental web platforms, particularly for visual stimuli. This variability also suggests that online studies may not achieve a similar level of precision as lab-based studies. Moreover, since the data are contributed anonymously and without supervision, online data could be compromised by dishonest participants with low or questionable motivation [36].

In fact, several comparative studies report only a moderate correlation between web-based cognitive performance and its paper and pencil alternatives across different cognitive tasks and populations [32,36,67]. For instance, Backx et al [34] compared cognitive data obtained from the CANTAB platform, which was collected using unsupervised web-based and lab-based administration. Intraclass and bivariate correlations showed that several key performance indices (errors, correct trials, and response sensitivity) were highly comparable across the two settings, with intraclass correlation coefficients ranging from ρ=0.23-0.67. However, participant reaction times (RTs) were off-task significantly and consistently slower for web-based assessments, and none of the 5 RT measures that were assessed met the full criterion for comparability across settings, namely, reliability, equivalence, and agreement. Further, in the online setting, over 90% of participants reported being distracted across 5 different cognitive tasks, as compared to none in lab settings, and 2 online participant data sets were excluded due to a high number of errors on the spatial working memory (SWM) task. These statistics suggest that a poor test environment and miscomprehension of instructions could affect participant performance in online settings. Indeed, previous research suggests that a lack of incentive can make participants careless in their responses or even deceptive, as participants’ identity or behavior cannot be actively monitored [68]. Further, online participants may show lower task engagement by investing less time and focus on reading task instructions, leading to higher dropout rates than in laboratory settings [69,70]. Therefore, with current unsupervised web-based testing protocols, there appears to be a trade-off between data quantity (diversity and ease of collection) and data quality [37,66,71]. Given these known pitfalls in online testing, along with increased efforts toward standardizing best practices in online task administration, data quality indices, and reporting benchmarks (eg, Feenstra et al [72]), this study addresses a timely need to develop better test protocols and data quality assurance frameworks for web-based cognitive testing, specifically addressing issues with online participant engagement.

### Data Quality Assurance Framework for Web-Based Cognitive Testing

To assess data quality, it is first important to establish the indices and benchmarks by which data quality will be measured. On these points, there is currently no clear consensus. General statistics on participant noncompletion, data attrition, and technical issues show that it is common to exclude data from participants who encounter technical difficulties, display dishonesty, or fail to complete the assigned tasks [71,73-75]. However, a more sensitive test of data quality pertains to the “usable fraction” of data that remains after task-specific exclusion criteria for data cleaning have been applied. One common index used for data exclusion is RT since responses that are too fast (or too slow) are likely to reflect participant inattentiveness or task disengagement. Depending on the stimuli presented and the complexity of task demands, participant response latencies in cognitive tasks mostly vary between 400 milliseconds to 2000 milliseconds [76]. However, for web-based tasks, there is an additional (technical) source of variability to the measured response times. Collecting response latencies from many (eg, hundreds) individual trials requires a software program to be installed on the participant’s computer (ie, client-side), to present the stimuli and collect response latencies locally. This reduces the temporal variation introduced by communication across networks and server response times if each response must be sent over the network connection back to the server to be recorded. Several client-side technologies have been used to create such programs, with perhaps the most popular being JavaScript, Java, and Flash [76].

However, most of these programs introduce a small but variable delay in the recorded response times. In addition to the program itself, this delay is influenced by the computer’s operating system, browser, hardware quality, and any background programs that may be running. For example, when Schubert et al [76] measured standard automated response times (ie, robot detection of a simple visual stimulus) natively using DMDX software and a keyboard, the mean response time was 68.24 milliseconds (SD 3.18). These mean latencies were higher when other programs were used, for instance, E-prime (84.58 milliseconds, SD 6.25) and Superlab (98.18 milliseconds, SD 4.17). Interestingly, mean response latencies were highly comparable for the web version of Inquisit (66.21 milliseconds, SD 2.74). When comparing human response times on a Stroop Task, Schubert et al [76] reported that DMDX-recorded responses were significantly faster (mean 551.98 milliseconds, SD 201.38) than Flash-based web software ScriptingRT (mean 631.63 milliseconds, SD 243.42), although the measured Stroop effect (difference in response latency between incongruent and congruent trials) was similar. Therefore, web-collected response latency data should be carefully handled as the measured timings may be impacted by both psychological (eg, participant distraction and inattentiveness) and technical factors, although the latter issue is ameliorated to some extent by newer and better online experimental platforms.

Some web-based studies implement a hard cut-off to exclude response latencies past a particular threshold to optimize data quality. For instance, Kim et al [77] excluded outlier responses that were faster than 300 milliseconds or slower than 5000 milliseconds in a psycholinguistic task they employed. However, these excluded trials represented a mere 0.75% of their data (for both lab-based and web-based cohorts) which could either suggest superb data quality or that their latency criteria were too lax for the particular task. Similarly, Eisenberg et al [55] excluded participants whose median response latencies were shorter than 200 milliseconds across a wide range of cognitive tasks. They also implemented three additional quality checks: (1) <25% omitted (missed) responses, (2) >60% task accuracy, and (3) no single response given >95% of the time. However, although rates for participant noncompletion and multiple task failure were reported (Table 1), the number of trials and data sets that failed their other response latency, omission, and distribution quality checks were not reported. Further, for some tasks (Stop Signal, probabilistic selection, and two-step decision tasks), the data sets had between 10% to 30% missing values that were identified through additional quality control (manipulation) checks. Adding this figure to the reported 21% of data exclusions suggests that the actual fraction of “usable data” could be as low as 50% on some web-based tasks.

Table 1 provides examples of other data exclusion statistics for analogous lab-based and web-based cognitive studies. These statistics report participant completion/dropout rates rather than trial-level data quality indices. Studies by Hicks et al [79] and Ruiz et al [80], who administered matched sets of working memory and declarative memory tasks in lab-based and online conditions, show a clear and consistent trend toward a higher rate of data exclusion and noncompletion for web-based testing, with typically 15% to 20% more online participants excluded for noncompletion (dropout) and technical issues. Participant dropout issues appear to be particularly exacerbated for online cognitive training studies, with one study reporting that 32% (80/249) of initially recruited healthy older adult participants eventually withdrew from their 12-week cognitive flexibility web-based training study [81]. However, this brief scan of the literature also highlights that, except for a few studies (eg, Backx et al [34]), little is reported about what occurs during online experimental testing or about the computing and web capabilities of participants, and few benchmarks exist for identifying and removing poor quality web-based data, beyond crude RT thresholds and major task failures. There is a clear need to develop standardized data quality indices that web-based studies should collect and report, including the recommended benchmark(s) for these indices.

**Table 1:** Examples of data exclusion statistics reported for lab-based and web-based cognitive studies.

Study type and citation		Task(s)	Data excluded
**Lab-based**			
	Kim et al [77]	Lab, psycholinguistic task	5/42 (11.9%) participants excluded for high error rates or being outside demographic. Reaction time outlier removal=0.75% of total data
	Von Gunten et al [82]	Lab, inhibition tasks (antisaccade, go/no go, and Stop Signal)	37/463 (7.99%) participants excluded
	Backx et al [34]	Lab, CANTAB^a^ tasks^b^	No exclusions, no distractions reported
	Hicks et al [79]	Lab (experiments 1 and 3), working memory tasks	Experiment 1: 0/58 (0%) participants excluded, although 10% of participants reported cheating; experiment 3: 10/112 (8.9%) participants excluded due to excessive missing data
	Ruiz et al [80]	Lab, working memory^c^, nondeclarative/declarative memory tasks	(a) OSpan^d^, 0% excluded; (b) MLAT5^e^, 0% excluded; (c) CVMT^f^, 1/50 (2%) participants excluded
	Baniqued et al [78]	Cognitive video training	27/219 (12.3%) participants excluded or withdrew
**Web-based**			
	Kim et al [77]	Online, psycholinguistic task	3/39 (7.7%) participants excluded for high error rates or being outside demographic. Reaction time outlier removal=0.75% of total data
	Eisenberg et al [55]	Online (using Amazon Turk), inhibition tasks (go/no go, Stop Signal)	102/662 (15.4%) participants excluded for noncompletion of task battery; 38/560 (6.8%) participants further excluded for failing 4 or more tasks
	Backx et al [34]	Online, CANTAB tasks	2/18 (11.1%) participants excluded, high SWM^g^ errors; distractions: 16/18 participants for PAL^h^, ERT^i^, OTS^j^, and PRM-I^k^; 17 participants for SWM, RVP^l^, PRM-D^m^
	Hicks et al [79]	Online (experiments 2 and 4), working memory tasks	Experiment 2: 12/100 (12%) participants excluded for failure to complete test battery within 24 hours; Experiment 4: 28/112 (25%) participants excluded due to noncompletion of task battery
	Ruiz et al [80]	Online, working memory, nondeclarative/declarative memory tasks	(a) OSpan, 7/50 (14%) participants excluded; (b) MLAT5, 8/15 (16%) participants excluded; (c) CVMT, 10/50 (20%) participants excluded
	Buitenweg et al [81]	Cognitive flexibility training	91/249 (36.5%) participants excluded for not meeting criteria (N=11) or withdrew from study (N=80)

^a^CANTAB: Cambridge Neuropsychological Test Automated Battery

^b^CANTAB tasks include SWM, PAL, ERT, OTS, PRM-I, RVP, and PRM-D.

^c^Memory tasks include OSpan, MLAT, and CVMT.

^d^OSpan: automated operation span task.

^e^MLAT: modern language aptitude test.

^f^CVMT: continuous visual memory task.

^g^SWM: spatial working memory.

^h^PAL: paired associates learning.

^i^ERT: emotion recognition task.

^j^OTS: one touch stockings of Cambridge.

^k^PRM-I: pattern recognition memory-immediate.

^l^RVP: rapid visual processing.

^m^PRM-D: pattern recognition memory-delayed.

### A New Supervised Online Method: Remote Guided Testing

In the preceding sections, we discussed the promise of web-based cognitive testing, specifically its scalability and reach, and the current challenges for data quality assurance. In part, questions over experimental rigor and data quality have arisen due to the unsupervised nature of web-based testing [36,37,66]. Without supervision, experimenters have no control over (or insight into) the test environment and no way to monitor participant performance, deter dishonesty, or influence participant motivational and attentional states during task performance. Further, even genuinely motivated participants may struggle with tasks that have complex instructions and misunderstand what is required of them, leading to wasted effort and unusable data. Finally, without a human experimenter on hand to troubleshoot problems, participants experiencing technical issues may become frustrated and stressed, leading to poorer motivation and performance. To bridge this gap, we propose here a new method of supervised online data collection, RGT. This hybrid method marries the convenience and reach of online web-based testing with the enhanced rigor and quality control of in-person lab-based data collection. The addition of a supervisory component, including greater environmental control, aims to mitigate data quality degradation and attrition due to psychological or technical factors.

The RGT method simulates lab-based experimental testing via a video conferencing platform. Similar to in-person testing, the experimenter arranges to meet the participant online at a specific date and time and guides the participant virtually through each step of the experimental process. This includes obtaining informed consent, providing technical support for software installation, troubleshooting problems, monitoring performance, providing feedback where appropriate, and debriefing. The experimenter also helps the participant to optimize their test environment (including lighting, sound, and minimizing distractions) and collects detailed data about the hardware, software, and web capabilities of the participant. Additionally, the remote tester can schedule comfort breaks (for toilet trips, food or drink, rest, exercise) so as not to affect test delivery or data collection adversely. This method is novel in its holistic approach as it provides a fully supervised and interactive online test experience, which to our knowledge has not been reported before for web-based cognitive testing.

To provide a deeper evaluation of the RGT method on data quality, we measure and report 3 trial-level data quality indicators across a range of web-based cognitive tasks: (1) missed responses, (2) data exclusions (at both trial and participant levels), and (3) RTs. To ensure close comparability and to isolate the effect of test modality, participants completed identical web-based versions of each cognitive task either in-person in a psychology lab or at home via RGT. In both conditions, participants received expert supervision while they completed a range of cognitive tasks assessing executive function (cognitive flexibility, inhibition, and working memory) and learning. While most of these tasks rely on measures of accuracy, we specifically included tasks with RT-dependent outcome measures, such as the Stroop Task [20] and the Stop Signal Task [21,22]. Given that there are well-quantified effects of web-based testing in terms of slower participant RTs on cognitive tasks [34,76], we assessed if (and the extent to which) these differences could be ameliorated through greater supervisory and environmental control. Finally, to increase the generalizability of our findings, 3 different web-based experimental platforms were tested; CANTAB, Inquisit, and i-ABC. We hypothesized that the inclusion of supervision via the RGT method would yield high fidelity cognitive data that match lab-collected cognitive data in all measured indices of data quality and task performance (including RTs).

## Methods

### Participants

A total of 85 healthy Singaporean young adults participated in the study and contributed data face-to-face (F2F; n=41) and via RGT (n=44). A further 4 RGT and 5 F2F participants had initially expressed interest but subsequently withdrew from the study. Data from these participants were not included in any analyses. All participants were native English speakers, reported no history of clinically diagnosed mental illness or developmental difficulties, and had normal or corrected hearing and vision. Recruitment was conducted through online advertisements in social media outlets and through the University’s recruitment channel. The demographic information for both groups is detailed in Table 2. A two-tailed *t* test confirmed that there was no significant difference in age between groups (*t*_83_=–1.29, *P*=.20), and gender, ethnicity, education, and income distributions were also similar.

All 85 participants attended and completed their scheduled testing session(s). None of them withdrew midway through their session(s). All 44 (100%) participants in the RGT group completed all 10 computerized tasks on web-based platforms. However, only 22 (53.7%) F2F participants completed all the computerized tasks on web-based platforms (17 females and 5 males; mean age 21.06 years, range=18.11-26.68 years, SD 2.09 years). The remaining 19 (46.3%) F2F participants were tested before COVID-19 lockdown restrictions and therefore only completed the 3 i-ABC tasks, vocabulary, and Digit Span tasks in a format similar to the other participants. The other Inquisit tasks (Trails, Stop Signal, and Stroop) had either been completed on paper or using a different (offline) platform, and the CANTAB tasks were not administered. As these task-related differences could have generated performance differences, for consistency, only the data from the i-ABC tasks, vocabulary, and Digit Span were analyzed for these 19 (46.3%) F2F participants.

**Table 2:** Summary of participant demographics by testing modality.

Demographic variable		Modality (group)				
		F2F^a^ (n=41)		RGT^b^ (n=44)		Total (N=85)
**Age (years)**						
	Mean (SD)	21.54 (2.26)	22.14 (2.05)		21.85 (2.16)	
	Range	18.11-29.22	18.51-26.83		18.11-29.22	
**Gender, n (%)**						
	Female	29 (70.7)	33 (75)		62 (72.9)	
	Male	12 (29.3)	11 (25)		23 (27.1)	
**Ethnicity, n (%)**						
	Chinese	34 (82.9)	36 (81.8)		70 (82.4)	
	Malay	4 (9.8)	6 (13.6)		10 (11.8)	
	Indian	2 (4.9)	2 (4.5)		4 (4.7)	
	Not reported	1 (2.4)	0 (0)		1 (1.2)	
**Income by dwelling, n (%)**						
	Lower	13 (31.7)	16 (36.4)		29 (36.3)	
	Higher	24 (58.5)	27 (61.4)		51 (63.7)	
	Not reported	4 (9.8)	1 (2.3)		5 (5.9)	
**Highest education level, n (%)**						
	Secondary School	27 (65.9)	23 (52.3)		50 (58.8)	
	Bachelor’s Degree	12 (29.3)	16 (36.4)		28 (32.9)	
	Not reported	2 (4.9)	5 (11.4)		7 (8.2)	
**Handedness, n (%)**						
	Right-handed	38 (92.7)	42 (95.5)		80 (94.1)	
	Left-handed	2 (4.9)	2 (4.5)		4 (4.7)	
	Not reported	1 (2.4)	0 (0)		1 (1.2)	

^a^F2F: face-to-face.

^b^RGT:remote guided testing.

### Equipment

For the F2F group, experimental testing was conducted in a psychology lab using a standard testing laptop (HP ProBook 430 G2/G3, Intel Core i7 2/2.4GHz, 8 GB RAM, 500 GB HDD +256 GB SSD, 13.3” display) running Windows 10 OS (Microsoft Corporation), with a wired mouse. For the RGT group, sessions were completed at home using participants’ personal laptops or desktops that had to meet certain minimum requirements (Multimedia Appendix 1). To assess the actual quality of their computing hardware and web capability, all RGT participants completed an equipment questionnaire (Multimedia Appendix 2).

### Procedure

**Figure 1:**
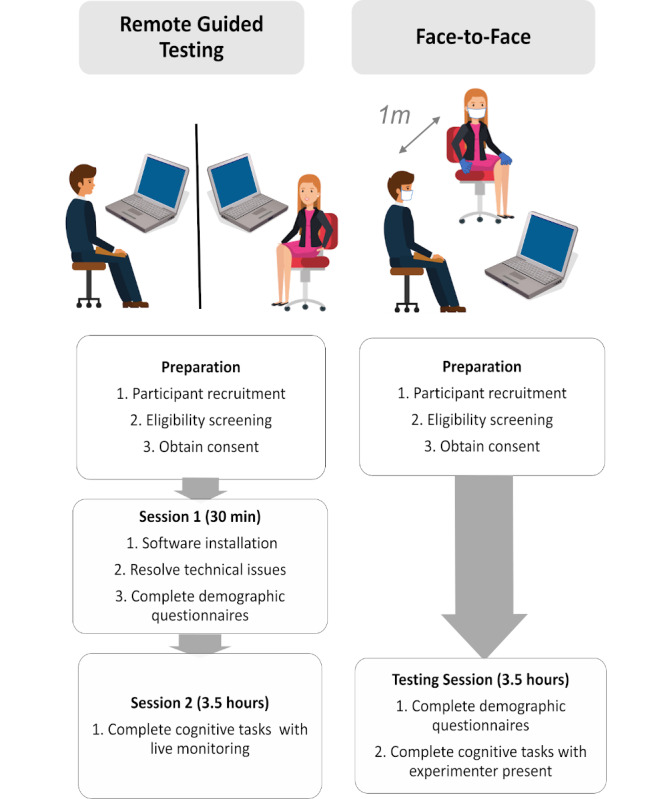
Overview of remote guided and face-to-face testing processes.

A standard operating procedure (Multimedia Appendix 1) was followed to ensure standardized methodology and task delivery for participants in the F2F and RGT groups. Prevailing COVID-19 precautions such as mask-wearing, temperature-taking, and checking of travel/quarantine history were also applied. The study protocol was approved by the NTU Ethics Institutional Review Board (IRB-2020-02-001). In brief, F2F participants completed 1 single in-person testing session lasting 3.5 hours whereas, RGT participants completed 2 separate online video-conferencing sessions, which were conducted via secure Microsoft Teams or Zoom meetings. Both online sessions were video recorded and lasted 4 hours in total. During the first 30-minute install and set-up session, participants were guided by the experimenter to download, install, and test all necessary software. The testing environment was assessed to provide recommendations for minimizing noise and disruption (Multimedia Appendix 3, see testing environment checklist), and computing and input devices were recorded (Multimedia Appendix 2). During the second 3.5-hour session, RGT participants performed all computerized tests under the supervision of the experimenter, who remained online throughout the session (with their video off/muted where appropriate). Five different task orders were generated to ensure that no 2 tasks from the same cognitive domain were administered consecutively. The respective procedures are summarized in Figure 1.

### Tasks

#### Overview

In total, participants completed 10 online experimental tasks assessing aspects of executive functioning (cognitive flexibility, working memory, and inhibitory control), learning, and verbal intelligence. These tasks were delivered using 3 different experimental web platforms: i-ABC [83], Inquisit 5 (Millisecond Software), and CANTAB (Cambridge Cognition); or delivered verbally by the experimenter, as summarized in Table 3. Both groups also completed a short online demographics questionnaire.

Table 3 provides an overview of the various delivery platforms that were used in this study and the full set of tasks. Checkmarks indicate the respective platform on which the task was administered.

**Table 3:** Summary of experimental tasks administered and respective delivery platforms.

Domains and tasks		Delivery platform			
		i-ABC	CANTAB^a^	Inquisit	Verbal
**Cognitive flexibility**					
	WCST^b^	✓	—^c^	—	—
	PR^d^	✓	—	—	—
	TMT^e^	—	—	✓	—
	IED^f^	—	✓	—	—
**Working memory**					
	SWM^g^	—	✓	—	—
	WAIS-IV BDS^h^	—	—	—	✓
**Inhibition**					
	Stroop Task (Stroop)	—	—	✓	—
	SST^i^	—	—	✓	—
**Learning**					
	SL^j^	✓	—	—	—
**Verbal IQ^k^**					
	WASI-II^l^ vocabulary (vocab)	—	—	—	✓

^a^CANTAB: Cambridge Neuropsychological Test Automated Battery.

^b^WCST: Wisconsin Card Sort Test.

^c^Empty cells indicate that the particular task was not administered via the specific delivery platform.

^d^PR: probabilistic learning and reversal.

^e^TMT: trail making task.

^f^ED: intra-extra dimensional set shift.

^g^SWM: spatial working memory.

^h^WAIS-IV BDS: Weschler Adult Intelligence Scale–Fourth Edition Backwards Digit Span.

^i^SST: Stop Signal Task.

^j^SL: structure learning.

^k^Q: intelligence quotient.

^l^WASI-II: Weschler Abbreviated Scale of Intelligence–Second Edition.

#### i-ABC Platform

Three experimental tasks were administered on the i-ABC platform [83]. The Wisconsin Card Sort Test [15,16] and the probabilistic reversal task [84] were measures of cognitive flexibility, whilst the Structure Learning task [19,83] assessed statistical learning. The i-ABC website enabled the administration of the 3 tasks on a platform that simulated playing a “space-themed” video game, and participants earned points for completing the tasks. Detailed task descriptions and performance indices are provided in Multimedia Appendix 4 (see subsections 1-3).

#### Inquisit 5 Web Platform

Computerized versions of the Stroop Task, Stop Signal Task, and the Trail Making Task were hosted and administered on the Inquisit 5 web player by Millisecond software [85]. The software was downloaded before the session, and when each task link was opened, participants were prompted to key in their unique ID before launching into full-screen mode. The display dimensions of the task stimuli were standardized and automatically adjusted by the software according to the computer physical screen display size. Detailed task descriptions and performance indices are provided in Multimedia Appendix 4 (see subsections 4-6).

#### CANTAB Platform

The intra-extra dimensional (IED) set shift task and spatial working memory task were both administered as part of CANTAB [26,27,86]. Detailed task descriptions and performance indices are provided in Multimedia Appendix 4 (see subsections 7-8).

#### Verbal Delivery

The vocabulary subtest of the second edition of the Wechsler Abbreviated Scale of Intelligence (WASI-II) [28] and the Backwards Digit Span subtest from the fourth edition of the Weschler Adult Intelligence Scale (WAIS-IV) [29] were administered via verbal delivery to assess verbal intelligence and verbal working memory respectively. Detailed task descriptions and performance indices are provided in Multimedia Appendix 4 (see subsections 9-10).

### Data Quality Indicators

#### Missed Trials

For tasks involving a response within a specified time limit, the number of missed trials was calculated. If a participant did not enter a response within the specified time limit for a trial, this was considered a missed trial. As some tasks (eg, Stroop) required a response before proceeding, this index was not available for these tasks.

#### Data Exclusion

Participant data could be excluded either at the trial level or at the task level (ie, all participant data removed for that task).

##### Trial-Level Exclusions (Outliers)

Single trials were excluded if the RT on that trial was outlying (either too fast or too slow). Referencing previous research using similar tasks [55,77], response times faster than 300 milliseconds are generally deemed to indicate participant inattentiveness or a failure to fully process the stimulus on that trial. Conversely, response times that are greater than SD 2.5 of the response time distribution are also generally considered to be outliers, indicating failures of attention. The F2F RT distribution was used to set a fixed threshold for both groups to ensure that the basis for identifying slow outlier response times was consistent and provided a fair basis of comparison of data quality. Specifically, slow outliers were defined as RTs >2.5 SDs above the mean of the F2F distribution for each task. Any trials with RTs slower than this threshold (for both F2F and RGT participants) were considered outliers and removed.

As an exception for the Stop Signal Task, and with reference to Verbruggen et al [22], the first trials for each of the 3 blocks were removed as participants were not expected to have fully engaged with the task at this early stage. Additionally, RTs under 300 milliseconds were not removed for the Stop Signal Task as participants were required to provide a speeded response, and failures of inhibition were of core interest. As the CANTAB web platform did not provide trial-level data, no trials were excluded for the IED or SWM tasks. Since the verbal delivery tasks (WASI Vocabulary and Backwards Digit Span) were administered manually by the experimenter in both the F2F and RGT settings, they were not subject to trial-level exclusions.

##### Task-Level Exclusions

At the task level, participant data were excluded for either technical or performance reasons. Data were excluded for technical reasons if the participant experienced difficulties with the experimental platform, equipment, or testing environment during that task. Task-level performance exclusions occurred if the participant’s total number of missed and excluded trials was >25% of all trials for that task (see previously discussed criteria for trial-level exclusions).

#### Reaction Times

The final index of data quality was the mean RT (by participant) of the remaining included trials. This was used as a data quality indicator because previous studies have indicated that mean RTs may be more variable/longer during web-based delivery of experimental tasks [34,76]. As the CANTAB web platform did not provide trial-level data, this RT index was not available for the IED or SWM tasks.

## Results

### Technology Profile of Remote Guided Participants

**Figure 2:**
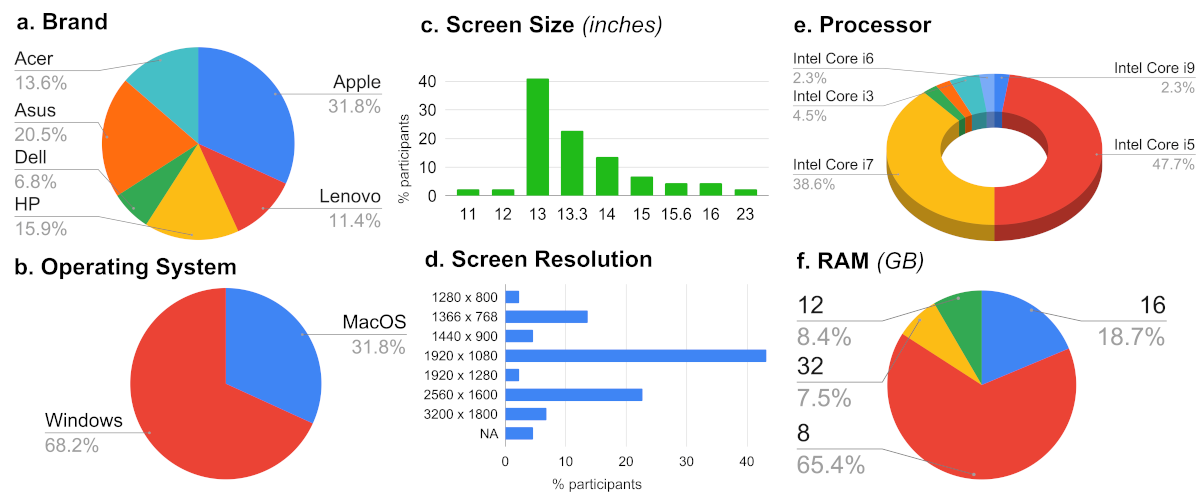
Hardware specifications for remote guided participants (total N=44), including computer (a) brand; (b) operating system; (c) screen size (in inches) (d) screen resolution (in pixels); (e) processor and (f) RAM (in GB).

**Figure 3:**
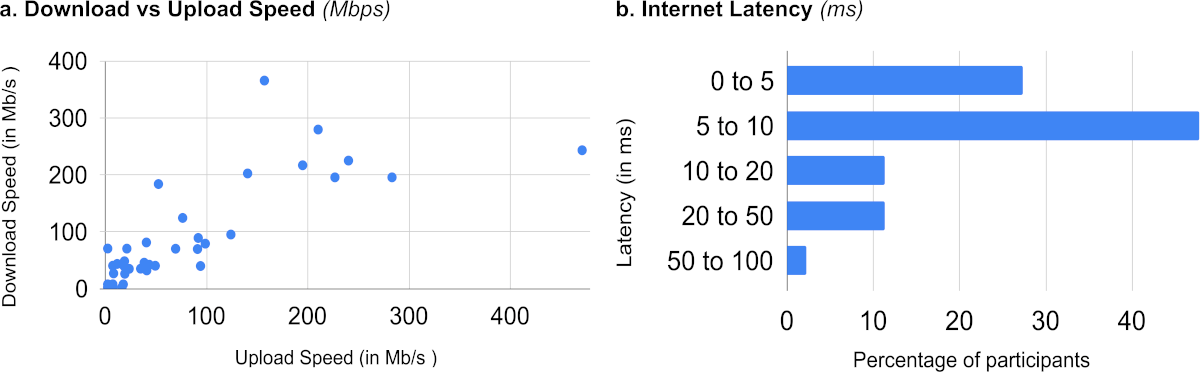
Web capability for remote guided participants (total n=44), including (a) internet download/upload speed (higher=better); and (b) internet latency (shorter=better).

Each RGT participant completed the experimental tasks at home using their personal computer and internet connection. Although all participants used equipment that met certain minimum standards as stated in the eligibility criteria (see Equipment in the Methods section), we wished to determine the actual range and quality of technology that was being used. As shown in Figures 2-3 and detailed in Table 4, the lab equipment was a close match to the hardware specifications reported by the RGT group (eg, Windows OS, Intel Core i7 processor, 13-inch screen, 1920 x 1080 resolution, approximately 8 GB RAM). In terms of web capability, most RGT participants had better internet download/upload speeds than the F2F group (mean of 77.9/70.4 Mb/s vs 44.6/48.1 Mb/s) but slightly longer internet latencies (mean of 10.6 milliseconds as compared to 5 milliseconds).

**Table 4:** Summary of hardware and web capability specifications for remote guided participants, compared to the standard testing equipment used for the face-to-face group.

Hardware specifications			RGT^a^ n/mean, (%/SD)		F2F^b^ standard
**Brand**					
	Acer	14 (13.6%)		HP Probook	
	Apple	6 (31.8%)		—^c^	
	Asus	9 (20.5%)		—	
	Dell	3 (6.8%)		—	
	HP	7 (15.9%)		—	
	Lenovo	5 (11.4%)		—	
**Operating system**					
	Windows	30 (68.2%)		Windows 10	
	Mac OS	14 (31.8%)		—	
**Processor**					
	Intel Core i3	2 (4.5%)		Intel Core i7 2/2.4ghz	
	Intel Core i5	21 (47.7%)		—	
	Intel Core i6	1 (2.3%)		—	
	Intel Core i7	17 (38.6%)		—	
	Intel Core i8	1 (2.3%)		—	
	Intel Core i9	1 (2.3%)		—	
	Other	1 (2.3%)		—	
RAM (GB)			9.73 (4.35)		8.0
Total hard disk space (GB)			417 (229)		500 HDD (+256 SSD)
Free hard disk space (GB)			270 (223)		108
Screen size (inches)			13.8 (1.74)		13.3
**Screen resolution**					
	1280 x 800	1 (2.3%)		1920 x 1080	
	1366 x 768	6 (13.6%)		—	
	1440 x 900	2 (4.6%)		—	
	1920 x 1080	19 (43.2%)		—	
	1920 x 1280	1 (2.3%)		—	
	2560 x 1600	10 (22.7%)		—	
	3200 x 1800	3 (6.8%)		—	
	Unspecified	2 (4.6%)		—	
**Input devices**					
	Mouse (wireless)	27 (61.2%)		Wired mouse	
	Mouse (wired)	15 (34.1%)		—	
	Mouse (integrated)	2 (4.6%)		—	
	Keyboard (wireless)	2 (4.6%)		Integrated keyboard	
	Keyboard (integrated)	42 (95.5%)		N/A^d^	
	Webcam (integrated)	43 (97.7%)		Integrated webcam	
	Webcam (separate)	1 (2.3%)		N/A	
	Microphone (integrated	35 (79.6%)		Integrated microphone	
	Microphone (separate)	9 (20.5%)		N/A	
**Web Capability**					
	download speed (Mb/s)	77.9 (88.6)		44.6	
	Upload speed (Mb/s)	70.4 (96.1)		48.1	
	Internet latency (ms)	10.6 (12.3)		5	
**Web browser**					
	Google Chrome	38 (86.4%)		Google Chrome	
	Mozilla Firefox	5 (2.3%)		N/A	
	Safari	1 (11.4%)		N/A	

^a^RGT: remote guided testing.

^b^F2F: face-to-face.

^c^We used one set of standard equipment for testing the F2F participants, hence there is only one value reported for each subheading under the F2F column.

^d^N/A: not applicable.

### Data Quality

#### Missed Trials

Table 5 shows the percentages of trials missed for each experimental task and group. To assess whether there was a difference in the number of missed trials across tasks as a function of testing modality, a general linear model (GLM) analysis with missed trials on each task (4 levels) as dependent (within-subjects) variables and modality (2 levels) as a predictor (between-subjects) variable was employed. Participants’ age and vocabulary standardized scores were entered as covariates in the model. Since Mauchly’s test indicated that the assumption of sphericity had been violated (*χ*^2^_5_=176; *P*<.001), degrees of freedom were corrected using Greenhouse-Geisser estimates of sphericity (*ε*=0.53). The results indicated no significant main effect of modality (*F*_1,56_=.61; *P*=.44; *η*^2^*P*=.01) and no significant interaction between modality and task (*F*_1.59, 89.3_=.44; *P*=.60; *η*^2^*P*=.01). Tukey HSD posthoc tests indicated that F2F and RGT participants did not differ on missed trials for any individual task (*P*>.99 for all pairwise comparisons). There were also no significant effects of age (*F*_1,56_=.52; *P*=.47; *η*^2^*P*=.01) and vocabulary (*F*_1,56_=.01; *P*=.91; *η*^2^*P*=.00).

**Table 5:** Summary of data quality indices for all tasks.

Delivery platform and task		(1) Missed trials (%), mean (SD)			(2) Data exclusion										(1) Reaction time (sec), mean (SD)			
					Trial level (%), mean (SD)					Task level (N), (tech/perf)								
		F2F^a^	RGT^b^		F2F		RGT		F2F			RGT		F2F			RGT	
**i-ABC**																		
	Wisconsin Card Sort Test (WCST)	0.73 (1.3)	1.02 (1.9)		3.50 (3.2)		4.92 (5.4)		0/0			0/ 0		1.33 (0.18)			1.39 (0.22)	
	Probabilistic learning and reversal (PR)	0.30 (0.6)	0.74 (1.5)		3.06 (3.1)		5.80 (5.9)		0/1			1/1		0.90 (0.16)			1.01 (0.21)	
	Structure learning (SL)	3.41 (2.6)	3.27 (2.6)		0.99 (0.7)		1.72 (3.5)		0/0			1/1		1.07 (0.15)			1.04 (0.16)	
**Inquisit**																		
	Color-Word Stroop	N/A^c^	N/A		3.38 (4.5)		3.28 (4.7)		0/0			1/1		0.84 (0.13)			0.87 (0.14)	
	Stop Signal Task (SST)	0.98 (1.8)	1.59 (3.3)		1.41 (2.4)		1.14 (2.7)		0/1			1/1		0.47 (0.08)			0.42 (0.09)	
	Trails A and B	0 (0)	0 (0)		0 (0)		0 (0)		0/0			2/0		40.9 (10.7)			40.4 (10.2)	
**CANTAB^d^**																		
	Intra/extra-dimensional set shift (IED)	N/A	N/A		N/A		N/A		0/0			0/0		N/A			N/A	
	Spatial working memory (SWM)	N/A	N/A		N/A		N/A		0/0			0/0		N/A			N/A	
**Verbal**																		
	Backwards Digit Span	N/A	N/A		N/A		N/A		N/A			0/0		0/0			N/A	
	WASI^e^ vocabulary	N/A	N/A		N/A		N/A		N/A			0/0		0/0			N/A	

^a^F2F: face-to-face.

^b^RGT: remote guided testing.

^c^N/A: not applicable.

^d^CANTAB: Cambridge Neuropsychological Test Automated Battery.

^e^WASI: Wechsler Abbreviated Scale of Intelligence.

#### Data Exclusion

##### Trial-Level Exclusions

Table 5 provides a full breakdown of data exclusions by experimental task and group. The data were analyzed using a GLM with excluded trials on each task (5 levels) as dependent (within-subjects) variables and modality (2 levels) as a predictor (between-subjects) variable to determine whether there was a difference in the overall percentage of excluded trials across tasks as a function of testing modality. Participants’ age and vocabulary standardized scores were entered as covariates in the model. Since Mauchly’s test indicated that the assumption of sphericity had been violated (*χ*^2^_9_=17.1; *P*=.047), degrees of freedom were corrected using Greenhouse-Geisser estimates of sphericity (*ε*=0.88). The results indicated no significant main effect of Modality (*F*_1,56_=2.1; *P*=.15; *η*^2^*P*=.04) and no significant interaction between modality and task (*F*_3.55, 198.5_=.37; *P*=.81; *η*^2^*P*=.01). Tukey HSD posthoc tests indicated that F2F and RGT participants did not differ on excluded trials for any individual task (*P*>.40 for all pairwise comparisons). There were also no significant effects of age (*F*_1,56_=1.97; *P*=.17; *η*^2^*P*=.03) and vocabulary (*F*_1,56_=1.74; *P*=.19; *η*^2^*P*=.03).

##### Task-Level Exclusions

As shown in Table 5, a total of 12 participant task-level data sets (F2F=2, RGT=10) were excluded from the analysis. Of these, 6 data sets were removed for technical reasons, and 6 were removed for performance reasons. Technical exclusions only occurred for the RGT group due to technical issues encountered during task administration (eg, OS compatibility, software/hardware issues, and environmental disruption). No data sets in the F2F group were excluded for technical reasons. For performance-related exclusions, recall that task-level data were excluded if the participant’s total number of missed and outlying trials was >25% of all trials for that task. Following these criteria, 2 participant task-level data sets were removed in the F2F group, and 4 participant task-level data sets were removed in the RGT group (a total of 6 datasets removed for performance reasons).

#### Reaction Times

Finally, we assessed whether the mean RTs of included trials differed as a function of testing modality (see Table 5 for group means). The data were analyzed using a GLM with RTs on each task (6 levels) as dependent (within-subjects) variables and modality (2 levels) as a predictor (between-subjects) variable. Participants’ age and vocabulary standardized scores were entered as covariates in the model. Since Mauchly’s test indicated that the assumption of sphericity had been violated (*χ*^2^_14_=176; *P*<.001), degrees of freedom were corrected using Greenhouse-Geisser estimates of sphericity (*χ*=0.37). The results indicated no significant main effect of modality (*F*_1,55_=.15; *P*=.70; *η*^2^*P*=.00) and no significant interaction between modality and task (*F*_1.86, 102.3_=.21; *P*=.24; *η*^2^*P*=.03). Tukey HSD posthoc tests indicated that F2F and RGT participants did not differ on RT for any individual task (*P*>.80 for all pairwise comparisons). There were also no significant effects of age (*F*_1,55_=.00; *P*=.98; *η*^2^*P*=.00) and vocabulary (*F*_1,55_=.14; *P*=.71; *η*^2^*P*=.00).

### Task Performance

**Figure 4:**
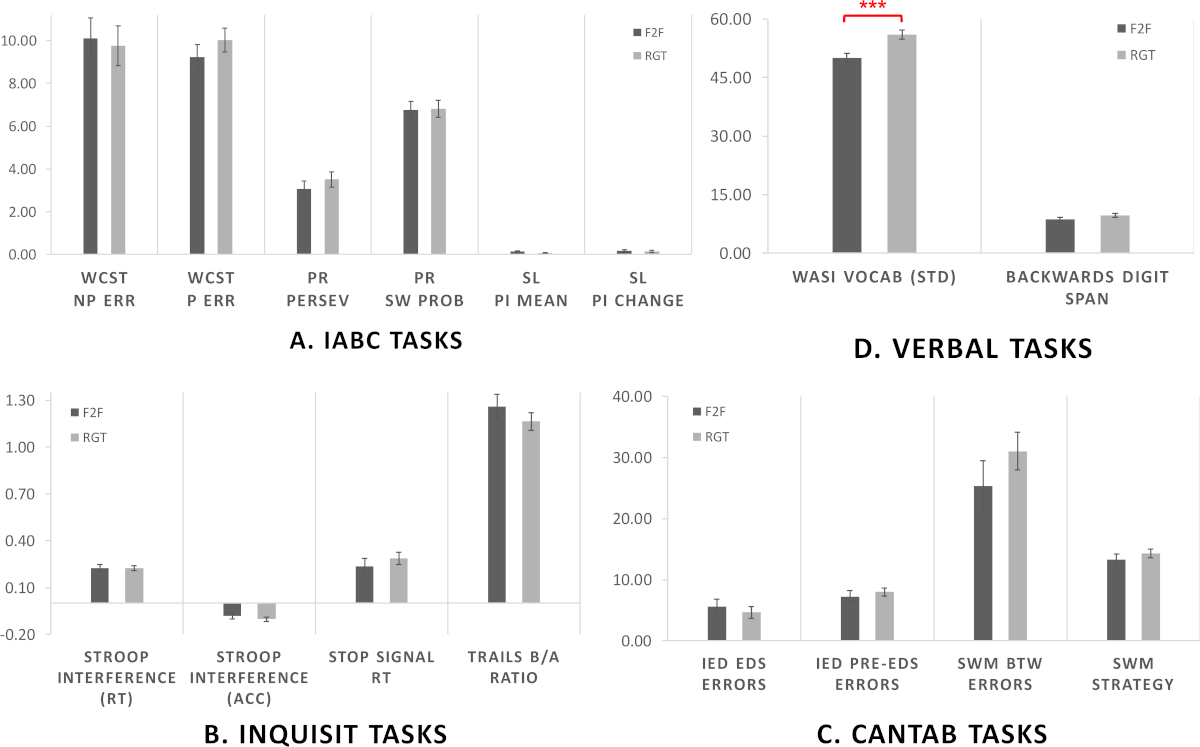
Plot of performance indices for (a) i-ABC; (b) Inquisit; (c) CANTAB and (d) Verbally delivered tasks. Face-to-face participants are shown in dark grey bars, remote guided participants are shown in light grey bars. Error bars indicate the standard error of the mean, ***P<.001.

The task performance indices were analyzed by delivery platform. Unlike the previous data quality measures of missed/excluded trials which were computed manually using simple and uniform criteria, these performance indices varied greatly in complexity and granularity (eg, spanning estimations of strategy, accuracy/error, and timing). Most of the performance indices were also automatically calculated by the delivery software using built-in criteria and assumptions. Accordingly, we analyzed task performance separately by delivery platform to allow us to detect any testing modality differences that emerged on some platforms and their tasks, but not others. Figure 4 and Table 6 show a full breakdown of participant performance by delivery platform, task, and test modality.

**Table 6:** Summary of task performance indices.

Delivery platform and task and performance index				Scores by group				GLM^a^ modality effects
				F2F^b^, mean (SD)		RGT^c^, mean (SD)		
**i-ABC**								Modality *F*_1,72_=.00; *P*=.96; Modality*Task *F*_1.20, 86.2_=.02; *P*=.73
	**Wisconsin Card Sort Test (WCST)**							
		Nonperseverative errors	10.1 (5.5)		10.2 (6.8)			
		Perseverative errors	9.3 (2.6)		10.0 (4.1)			
	**Probabilistic learning and reversal (PR)**							
		Perseveration	3.1 (1.7)		3.7 (2.8)			
		Switching probability	6.6 (2.5)		6.7 (2.6)			
	**Structure learning (SL)**							
		PI mean	0.15 (0.22)		0.06 (0.18)			
		PI change	0.18 (0.30)		0.16 (0.31)			
**Inquisit**								Modality *F*_1,58_=.74; *P*=.39; Modality*Index *F*_1.80, 104.3_=1.65; *P*=.20
	**Color-Word Stroop**							
		Interference (reaction time)	0.23 (0.10)		0.22 (0.11)			
		Interference (accuracy)	–0.08 (0.07)		–0.10 (0.09)			
	**Stop Signal Task (SST)**							
		Stop Signal reaction time	0.24 (0.19)		0.28 (0.27)			
	**Trails A and B**							
		Trails B/A time ratio	1.26 (0.43)		1.17 (0.31)			
**CANTAB^d^**								Modality *F*_1,59_=.02; *P*=.88; Modality*Task *F*_1,59_=.03; *P*=.86
	**Intra-extra dimensional set shift (IED)**							
		Extra dimensional shift errors	5.6 (7.2)		4.7 (5.2)			
		Pre-extra dimensional shift errors	7.3 (2.6)		9.6 (7.7)			
	**Spatial working memory (SWM)**							
		Between errors	25.3 (16.7)		32.3 (21.3)			
		Strategy	13.3 (4.6)		14.4 (4.1)			
**Verbal delivery**								Modality *F*_1,82_=16.6; *P*<.001; Modality*Task *F*_1,82_=7.57; *P*=.007
	WASI^e^ vocabulary (standardized score)			50.1 (7.2)		56.0 (7.6)		
	Backwards Digit Span (total score)			8.7 (3.1)		9.7 (3.2)		

^a^GLM: general linear model

^b^F2F: face-to-face.

^c^RTG: remote guided testing.

^d^CANTAB: Cambridge Neuropsychological Test Automated Battery.

^e^WASI: Wechsler Abbreviated Scale of Intelligence.

- **i-ABC:** The data were analyzed using a GLM with task (3 levels) and index (2 levels) as dependent (within-subjects) variables and testing modality (2 levels) as a predictor (between-subjects) variable to assess task performance across the three i-ABC tasks (Wisconsin Card Sort, probabilistic reversal and structure learning). Participants’ age and vocabulary standardized scores were entered as covariates in the model. Since Mauchly’s test indicated that the assumption of sphericity had been violated (task *χ*^2^_2_=78.7; *P*<.001), degrees of freedom were corrected using Greenhouse-Geisser estimates of sphericity (*ε*=0.60). The results indicated no significant main effect of modality (*F*_1,72_=.00; *P*=.96; *η*^2^*P*=.00) and no significant interaction between modality and task (*F*_1.20, 86.2_=.02; *P*=.92; *η*^2^*P*=.00). Tukey HSD posthoc tests indicated that F2F and RGT participants did not differ on performance for any individual task or index (*P*>.99 for all pairwise comparisons). There was a significant effect of age (*F*_1,72_=9.81; *P*=.003; *η*^2^*P*=.12) but no significant effect of vocabulary (*F*_1,72_=.02; *P*=.90; *η*^2^*P*=.00).
- **Inquisit:** To assess task performance across the three Inquisit tasks (Stroop, Stop Signal, and Trails), the data were analyzed using a GLM with index (4 levels) as dependent (within-subjects) variables and testing modality (2 levels) as a predictor (between-subjects) variable. Participants’ age and vocabulary standardized scores were entered as covariates in the model. Since Mauchly’s test indicated that the assumption of sphericity had been violated (task *χ*^2^_5_=65.9; *P*<.001), degrees of freedom were corrected using Greenhouse-Geisser estimates of sphericity (*ε*=0.60). The results indicated no significant main effect of modality (*F*_1,58_=.74; *P*=.39; *η*^2^*P*=.01) and no significant interaction between modality and index (*F*_1.80_,_104.3_=1.65; *P*=.20; *η*^2^*P*=.03). Tukey HSD posthoc tests indicated that F2F and RGT participants did not differ on performance for any individual task or index (*P*>.77 for all pairwise comparisons). There were no significant effects of age (*F*_1,58_=1.35; *P*=.25; *η*^2^*P*=.02) or vocabulary (*F*_1,58_=.11; *P*=.74; *η*^2^*P*=.00).
- **CANTAB:** Performance on the two CANTAB tasks (IED shift and SWM) was analyzed using a GLM taking task (2 levels) and Index (2 levels) as dependent (within-subjects) variables and testing Modality (2 levels) as a predictor (between-subjects) variable. Participants’ age and vocabulary standardized scores were entered as covariates in the model. The results indicated no significant main effect of modality (*F*_1,59_=.02; *P*=.88; *η*^2^*P*=.00) and no significant interaction between modality and task (*F*_1,59_=.03; *P*=.86; *η*^2^*P*=.00). Tukey HSD posthoc tests indicated that F2F and RGT participants did not differ on performance for any individual task or index (*P*>.51 for all pairwise comparisons). There was no significant effect of age (*F*_1,59_=.32; *P*=.57; *η*^2^*P*=.01) or vocabulary (*F*_1,59_=1.92; *P*=.17; *η*^2^*P*=.03).
- **Verbal delivery:** Finally, participants’ performance on the verbally delivered tasks (WASI Vocabulary and Backwards Digit Span) was assessed using a GLM with task (2 levels) as dependent (within-subjects) variables and testing modality (2 levels) as a predictor (between-subjects) variable. Only participant age was entered as a covariate in the model. Unlike all the previous tests, we observed a strong and significant main effect of modality (*F*_1,82_=16.6; *P*<.001; *η*^2^*P*=.17) as well as a significant interaction between modality and task (*F*_1,82_=7.57, *P*=.01; *η*^2^*P*=.08). Tukey HSD posthoc tests of the interaction indicated that F2F and RGT participants differed significantly on vocabulary performance (*P*<.001, RGT>F2F) but not on digit span (*P*=.83). There was no significant effect of age (*F*_1,82_=.96; *P*=.33; *η*^2^*P*=.01).

In summary, we observed no significant difference in task performance between F2F and RGT participants for any delivery platform or experimental task, with the notable exception of WASI Vocabulary, where RGT participants scored significantly higher than F2F participants on the task.

### Verbal Intelligence Analysis

To understand the source of this apparent difference in verbal intelligence, first, we assessed whether participants’ background could explain their differences in vocabulary performance. Accordingly, the categorical factors of gender (2 levels, male/female), ethnicity (3 levels, Chinese/Malay/Indian), education (2 levels, secondary/bachelors), home-dwelling (6 levels), and testing modality (2 levels, F2F/RGT), and the continuous variable of age were entered as predictors in a general regression model analysis, taking vocabulary score as the dependent variable. Overall, the model was significant (*F*_11,62_=2.89; *P*=.004; adjusted R^2^=0.22); however, the only significant predictor of vocabulary was testing modality (*β*=–0.47, SE 0.11, *t*=–4.15; *P*<.001). None of the other factors (age, gender, ethnicity, education, or dwelling) significantly predicted vocabulary scores (*P*>.25 for all). Therefore, group differences in verbal intelligence could not be explained by differences in participant background characteristics.

Next, we conducted further analyses on participants’ item-level responses. Recall that participants received 0 (for an incorrect or null response), 1 (for a partial response), or 2 points (for a full response) on each word item. We assessed whether superior performance in the RGT group was due to (1) knowledge of more words (ie, reaching a higher word item number) or (2) more complete description of words (ie, attaining a full score of 2 for a higher proportion of words). Unpaired two-tailed *t* tests conducted for each contrast revealed that RGT participants reached a significantly higher item number than F2F participants on average (F2F: mean 24.0, SD 1.3 and RGT: mean 25.3, SD 1.9; *t*_83_=–3.79; *P*<.001). However, a two-tailed *t* test showed that RGT participants also attained a full score on a higher proportion of items than F2F participants (F2F: mean 0.53, SD 0.23 and RGT: mean 0.63, SD 0.19; *t*_83_=–2.18; *P*=.03). Therefore, the item-level analysis supported both effects.

## Discussion

### Principal Findings

The COVID-19 pandemic has fundamentally changed the landscape of human psychological research and left in its wake a need for thoughtful recalibration of the balance between new remote ways of working and traditional lab-based research approaches. Never has there been greater urgency and impetus to shift toward web-based data collection methods. Yet data quality and assurance frameworks for online protocols—particularly for web-based cognitive measurements—are still lacking, and current published web-based studies vary greatly in their data quality monitoring and transparency. Therefore, we know surprisingly little about how web-based data sets differ from data collected in person, and significant questions remain regarding experimental rigor, reliability, and validity [36,37,66]. To help identify exactly how sources of unwanted participant variability may arise during online data collection and to mitigate these effects, we propose a new supervised online testing methodology, RGT. This hybrid method may offer a close alternative to traditional lab-based methods for collecting high-quality human cognitive data without requiring physical contact in the post-COVID “new normal” where many people now work from home.

Further, although we use RGT in a research context, our findings demonstrate there is no reason that the method could not be used clinically for neuropsychological assessments, particularly in situations where in-person meetings would be difficult or impossible. For example, people in wheelchairs or care homes may find it easier to be tested in their home environment, particularly during winter when daylight hours are short, and there can be significant weather deterrents to travel (eg, ice or snow). Therefore, there is wide potential for the RGT method to be used in tandem with traditional F2F methods across both clinical and nonclinical settings.

### RGT Data Quality

Three data quality indices were examined in cognitive test data collected via RGT and standard lab-based F2F methods: (1) missed trials, (2) data exclusion (both at the individual trial and participant level), and (3) RTs. The results showed that more participant data sets were excluded for technical reasons, such as hardware or software incompatibility issues, or in one case, environmental disruption in the RGT data set (n=6 across all tasks for RGT compared to none for F2F). However, RGT and F2F data sets did not differ on any of the other data quality indices of missed and excluded trials or on RT. The latter result is particularly relevant since previous web-based studies that have examined RT indices note significant and consistent lags in participant response time latencies during unsupervised web-based testing [34,76]. This indicates that experimenter supervision, even if only as a virtual presence, may be crucial for maintaining participant focus and attention on cognitive tasks, particularly when an expedient response is required. Additionally, the supervising experimenter was also able to quickly troubleshoot several common software and set-up problems that RGT participants experienced, which could have otherwise exacerbated the number of technical issues and data degradation.

It is well-established that the “experimenter effect” has a significant influence on participants’ motivation, mental state, performance, task engagement, and credibility during experimental studies [87,88]. It should also be noted that since F2F testing was also supervised, experimenter effects were likely to have been similar across groups, and in this case, apparently beneficial for task compliance. However, there are scenarios in which supervision may adversely affect participants’ cognitive and behavioral performance due to the social desirability effect and increased cognitive load [33,89,90]. For instance, Richman et al [89] reported decreased pressure to impress (social desirability effect) with the use of online-based settings. Therefore, the proposed RGT method may not be optimal for experimental paradigms that are sensitive to social desirability effects.

### RGT Task Performance

No significant differences in task performance were observed across all measures of executive function (cognitive flexibility, working memory, and inhibition) and learning, administered using 3 different experimental platforms (CANTAB, Inquisit, and i-ABC). However, we did observe a large and unpredicted difference in verbal intelligence (vocabulary) when measured in remote and in-person settings. Surprisingly, the RGT group scored significantly higher than the F2F group, and this effect could not be explained by differences in background characteristics (age, gender, ethnicity, or socioeconomic status). Detailed analyses of the item-level responses suggested that RGT participants produced correct definitions for a significantly higher number of words and also produced more fully elaborated responses to individual test items than F2F participants. This could be due to both F2F participants and the experimenter wearing facial masks and maintaining a physical distance of at least 1m (in compliance with prevailing COVID-19 guidance) throughout the experimental session in the lab. This could have influenced participants’ general willingness to communicate with the experimenter, consistent with data from a previous large-scale randomized control study indicating that mask-wearing by physicians during consultations negatively impacted doctor-patient communication, perceived empathy, and relational continuity [91]. Therefore, in clinical settings, remote testing methods not requiring the use of personal protective equipment such as masks may, in fact, be beneficial to reduce the communication barrier between experimenter and participant, thereby yielding improved performance on verbal tasks.

A strength of this study is that participants were of diverse and Asian origin (including Chinese, Malay, and Indian ethnicities), which addresses the Western skew in participant demographics that has characterized much of psychological research [42-47]. In this context, it is encouraging to note that web-based remote methodologies are suitable for these populations. However, most of the participants were highly educated university students whose attitudes, moral reasoning, beliefs, and social networks are known to differ significantly from that of nonuniversity educated counterparts [38]. Although the current study did not pertain specifically to social attitudes or phenomena, these factors may nonetheless have implicitly influenced data collection (eg, social desirability bias during experimenter monitoring, etc), limiting the broader generalizability of these findings to other populations.

### Toward a Data Quality Assurance Framework for Web-Based Cognitive Studies

Given the current societal momentum, we expect to see a continued rise in the number of cognitive studies conducted using web-based protocols. There exists, therefore, an urgent need for standardized protocols, data quality assurance indices, and benchmarks for the conduct and reporting of web-based cognitive studies. We take a step in this direction by making the standard operating protocol for our remote guided method freely available (Multimedia Appendix 1). We further report detailed information about participants’ technological capability and home environment, including the relevant survey instruments that were developed for this purpose (Multimedia Appendices 2 and 3). We define and report a detailed set of data quality indices, which include measures of trial-level variability (eg, missed responses, outlying responses, and RTs) as well as participant variability. We further distinguish between technical-related and performance-related issues and exclusions while providing in-depth descriptions of each. This level and form of reporting may help orient the field of web-based cognitive testing toward greater transparency, reliability, and replicability and also provide common metrics on which the data quality of different datasets may be compared [36,37,66].

### Limitations and Considerations for Selecting Test Methodology

**Figure 5:**
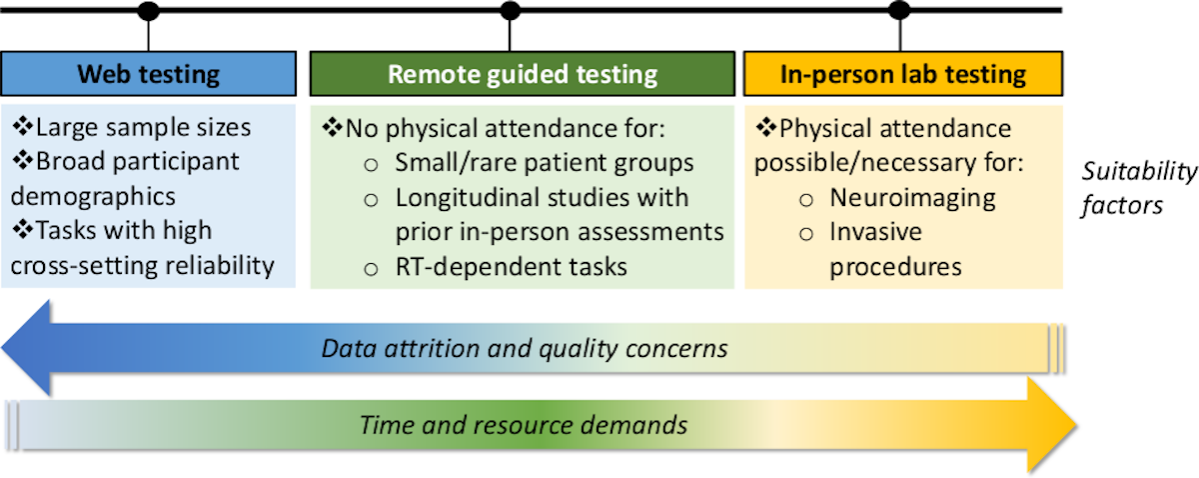
Summary of considerations for suitability of unsupervised, supervised web testing and in-person methodologies for cognitive testing. RT: reaction time.

Our results suggest that the RGT method yields high-quality cognitive data comparable to data collected in-person in the lab. However, this gain in data quality comes at the cost of additional manpower and time required for remote human supervision. In fact, compared to lab-based testing, the RGT method requires one additional set-up session (lasting 30 minutes) and therefore presents a greater time demand for both the experimenter and participant. This level of time investment may not be appropriate for large-scale studies that aim to test thousands of participants in a short period, although the inclusion of clear instructions for online tests and brief online tutoring (eg, using video clips) may improve the comprehensibility of instructions if the requisite research personnel are not available. As illustrated in Figure 5, one practical consideration when deciding on an appropriate test methodology is the trade-off between data quality and available time or resources. Purely unsupervised web-based testing has the attendant advantages of reaching large sample sizes with a broad demographic at a relatively low cost per head [34,49,54,56-58]. However, this may compromise data quality, comparability, replicability, and validity [61,62]. Therefore, implementing unsupervised web-based testing methods must be informed by the specific tasks to be used and their proven cross-setting reliability [34].

Another important consideration is the necessity and feasibility of in-person attendance at a physical location. Certain experimental protocols (eg, neuroimaging and invasive procedures) require in-person attendance due to the need for specific equipment or professional expertise. In these cases, in-person lab-based testing is the only option for data collection. However, in situations where physical attendance is not necessary or impossible (eg, during COVID-19 lockdown restrictions), RGT may be a viable alternative. The decision to adopt a method like RGT will be further weighted by considerations of group size and composition; for example, in clinical studies that involve high-risk or rare cohorts where maximization of individual data quality is important. Similarly, longitudinal studies that have used in-person lab-based cognitive tests at previous timepoints may prioritize cross-setting comparability, opting for supervised online methods that yield similar results to lab-based tests. Further, studies that include RT-dependent tasks (eg, Stroop and Stop Signal) may wish to use supervised online methods to ameliorate known reaction latency issues [76]. Finally, both supervised and unsupervised web-based methods require good internet connectivity and digital infrastructure for participants and research labs involved. The excellent web capability of RGT participants in the current study is indicative of Singapore having one of the highest levels of internet penetration in the world, recently estimated at 87% [92]. Therefore, while web-based testing would be highly feasible in countries like Singapore, this may be more challenging in countries with less well-developed digital infrastructure.

### Conclusions

The global COVID-19 pandemic has accelerated a move toward web-based cognitive testing, yet long-standing questions remain over the data quality and validity of web-based studies, compounding an urgent need to develop and implement data quality assurance frameworks for current and future online studies. Here, we propose a new supervised online testing methodology, RGT, and present data quality benchmarks for this new method. Across all measures of data quality and performance, the RGT method yielded data that was statistically equivalent to data collected in person in the lab. We conclude that the RGT methodology is robust and offers a viable alternative for collecting high-quality human cognitive data in both lab-based research and clinical contexts without requiring in-person physical attendance.

## Appendix

Multimedia Appendix 1 Standard operating protocols.

Multimedia Appendix 2 Equipment questionnaire (remote guided testing).

Multimedia Appendix 3 Testing environment checklist (remote guided testing).

Multimedia Appendix 4 Task descriptions and performance indices.

## Abbreviations

CANTAB: Cambridge Neuropsychological Test Automated Battery
CLIC: Centre for Lifelong Learning and Individualized Cognition
F2F: face-to-face
GLM: general linear model
HN: hybrid neuropsychology
IED: intra-extra dimensional
RGT: remote guided testing
RT: reaction time
SWM: spatial working memory
WASI: Wechsler Abbreviated Scale of Intelligence
