# Supplementary material for: A New Remote Guided Method for Supervised Web-Based Cognitive Testing to Ensure High-Quality Data: Development and Usability Study
Source: J Med Internet Res. 2022 Jan 6;24(1):e28368. doi: 10.2196/28368 (PMC8778570; doi:10.2196/28368)
Supplement: Multimedia Appendix 1 [file jmir_v24i1e28368_app1.pdf]

Multimedia Appendix 4: Standard Operating Protocols (SOPs)

1. Face-to-Face Testing

24 hours before the start of the experiment:

1. Email participant the following:
    a. Remind them that they are due to participate in this experiment and that they should report to you at least 15 minutes before the beginning of their timeslot.
    b. To comply with safety and precaution measures due to COVID-19, confirm with them if they have travelled in the past 2 weeks and if they have any symptoms of fever or any other health issues.
    c. Inform participant of the venue they should be reporting to.
    d. Remind participant that before they report to you (the experimenter), they will have to have their temperature taken and have their hands sanitised at the temperature station. We will ask and keep a record of their temperature at the start of the experiment.
    e. Lastly, remind them that if they feel unwell before starting the experiment, they will have to inform you (the experimenter) to cancel the testing session.
2. Create a contact log and fill in the name, date, time, temperature and phone number of each participant that you will be testing.

Actual Day of Experiment:

1. Sanitize all testing equipment (laptops, pens, blocks, etc) before testing.
2. Please also measure your own (the experimenter)'s temperature at the testing station and fill in your name, date, time, temperature beside the participant's entry in the temperature log.
3. Please wear a face mask throughout the experiment, keep a 1m distance whenever possible (e.g., sit behind the participant when they are doing the task).
4. Explain Information sheet and answer any questions they might have about the study. Obtain consent.
5. Remind them that since this is a long study, they will have frequent breaks in between.
6. Ask them to fill up the demographics questionnaire on iABC after they have created an iABC account following your instructions.
7. Conduct the tasks according to the randomization order.
8. Thank the participant and administer payment.
9. Check that all experimental data has been captured, logged and backed-up.
10. Sanitize all testing equipment (laptops, pens, blocks, etc) after testing.

2. Remote Guided Testing

Overview. The experimenter emailed each individual who expressed interest to check for eligibility and schedule the set-up and experiment sessions. Once confirmed, the experimenter assigned the individual a participant ID and sent a pre-setup documents zip folder containing the Information sheet and Consent form, the Equipment Guide *(available on request)* and the E-voucher Guide. Participants were requested to read through and digitally sign the consent form, as well as select their preferred E-voucher before sending these documents back to the experimenter prior to the first set-up session. In addition, each participant completed an online Equipment Questionnaire (Appendix A) on Qualtrics to ensure that they had met the specifications required for participation in the study. Upon submission, the experimenter checked the responses to ensure that the participant had the minimum specifications for successful task completion. These minimum requirements were:

1. For Windows users, a Windows 7 or 10 operating system (OS). For Mac users, OS Version 10.11.6 (El Capitan) or above;
2. At least 8 GB RAM;
3. Webcam and microphone;
4. Administrator rights to download and install software;
5. A reliable internet connection (latency speed not exceeding 50 ms);
6. Google Chrome, Safari or Mozilla Firefox web browsers installed;

One day before the set-up session, the experimenter sent a reminder email with a Microsoft Teams invitation link. The participant was instructed to download the Microsoft Teams desktop app for use across Sessions 1 and 2. Additionally, participants were requested to locate a quiet location (ideally a room with a closed door) where they would not be interrupted, and where sensitive video data would not be accidentally recorded. In the first session, the experimenter initiated a video call with the participant and once connected, video recording began. Upon meeting with the participant, the experimenter completed the Testing Environment checklist for Session 1 (Appendix B). The experimenter then reminded the participant to keep the laptop constantly charged, and to check for a strong and consistent internet connection. To minimise distraction, the participant was also reminded to switch off or put their phones on silent mode, and to place them face down. After ensuring an optimal testing environment, the participant was asked to share their screen via Teams to allow the experimenter to guide them on software installation and other tasks.

Session 1 Description:

In the first scheduled session, the experimenter guided the participant through 1) creating an iABC account, 2) downloading Inquisit Web and 3) completing a demographics questionnaire. Prior to Session 1, the experimenter sent an invitation link from the iABC platform to the participant's email address. The participant was instructed to access this link via their email, which prompted the participant to create an account on the iABC website. Once the iABC account had been successfully created, the experimenter then sent a link in the Teams chat for the participant to download Inquisit Web 5

(https://www.millisecond.com/download/inquisitweb5.aspx). Participants downloaded the installation file and followed the prompts to install the software. Once installation was complete, the experimenter reminded the participant not to delete Inquisit Web 5 off their OS for use in Session 2. Next, the experimenter asked the participant to complete the demographics questionnaire on the iABC website and explained the details of the questionnaire and how to complete it. Once the demographic questionnaire was completed, the experimenter confirmed the scheduled second session with the participant and ended the video call. The experimenter noted down any problems encountered during the first session and resolved any technical issues before the next session. The video recording of this session was uploaded to a secure location with restricted access for review purposes.

Session 2 Description:

One day before the second testing session, the experimenter sent a reminder email informing the participant of the scheduled time for Session 2, advising the participant to be in the same location as in Session 1 and to use the same Microsoft Teams invitation link that was used previously. At the start of Session 2, the experimenter checked that the participant's location was optimal and noted the observations in the Testing Environment Checklist for Session 2 (Appendix B). Similar to Session 1, participants were reminded to keep their laptops charging constantly, keep their phones switched off/ muted and face down and ensure that they had good Internet connectivity. Additionally, the experimenter requested the participant to turn off all running background apps and to mute desktop notifications as this would interfere with the proper completion of the tasks. The participant was also reminded to share the desktop with the experimenter via Teams as this ensured that the experimenter could monitor participants' progress across all tasks without them having to manually toggle between tasks and share them individually. Following these, the experimenter then conducted a sound quality check. To do this, the participant was first told to share audio via Teams by clicking on the "Share content" icon in Teams and selecting the option "Include computer sound". The experimenter then sent the participant a link to an audio clip consisting of beep tones (https://www2.iis.fraunhofer.de/AAC/ChID-BLITS-EBU-Narration441-16b.wav) and requested the participant to play the first 15 seconds of the clip to ensure that the volume was at a comfortable level. Once the sound check was over, the experimenter requested that the participant maximized screen brightness on his/ her computer for optimal performance and checked to see that the participant had a computer mouse in working condition. Lastly, the participant was reminded to fully maximise his/ her Web browser and to minimise the video screen panel before attempting all tasks.

After the aforementioned checks were completed, the experimenter guided the participant through the experimental tasks by sending each task link in the Teams chat and explaining the task instructions. The experimenter disabled the video function and muted the microphone after administering the task instructions prior to the start of each task to avoid interfering with participants' task performance. The experimenter monitored the participant's task progression and provided assistance if necessary throughout the whole of Session 2. The participant was allowed to take short breaks in between tasks to reduce eye strain and fatigue. All computerised tasks were completed by the participants independently, with the

experimenter intervening if there were any questions/ technical issues. However, for the WASI-II Vocabulary task, the experimenter enabled screen sharing with the participant to show the list of test items and conduct the task. For the Backwards Digit Span (BDS) task, the experimenter shared screen and system audio with the participant via Teams and set the audio to the maximum level, so that the participant could hear the spoken stimuli for the BDS task. Any technical issues encountered in Session 2 were noted down for future reference. Once the last task was completed, the experimenter issued an E-voucher or course credit, as appropriate, through email. This concluded Session 2. Similar to Session 1, the video recording of Session 2 was uploaded to a secure location with restricted access for review purposes.

Session 1 Checklist (for experimenters)

Before Session 1:

1. Email **Pre-setup document Zip folder** containing the Information Sheet and Consent Form, Equipment Guide, and E-voucher guide at least 3 days before the setup session. Upload the documents in a secure location with password-protected access.
2. Send a reminder email one day before scheduled set-up session.
3. Send iABC invitation link to participant's email address.
4. Create a Microsoft Teams chatroom using participant's email and include the invitation link to set up video call.
5. Once the participant has completed Equipment Questionnaire, check that all questions are completed and that participant meets minimum specifications to participate in the study.
6. Check that the consent form has been digitally signed and that the E-voucher selection guide is completed.

Setup (for experimenters):

1. Start video recording.
2. Inform participant that the video recording has started and kindly request them to share screen to allow us to guide them through installation.
3. Send Participant Guide for Session 1 *(available on request)*.
4. Once the participant has completed all pre-set up documents, check if he/she has any questions regarding the **Information Sheet**. Check for signed **Consent Form**.
5. If the participant has not completed the documents, go through the information sheet and request them to sign the consent form digitally.
6. Verify participants' answers on the **Equipment Questionnaire**. Check for:
    a. Computer specifications (check at least 8 GB RAM, HD)
    b. Physical Screen size and screen resolution
    c. Input devices (wireless or wired mouse/keyboard, webcam and microphone)
    d. Type of operating system- Windows 7 or 10. For Mac users, OS Version 10.11.6 (El Capitan) or above

    e. Type of web browser to be used (eg., Google Chrome (Recommended), Safari or Mozilla Firefox)
7. Record participant testing environment into **Testing Environment Checklist.**
    a. Lighting - ideally brightly-lit
    b. Sound - minimal background noise
    c. Room with closed door (recommended)
8. Download required software and test-run.
    a. Inquisit Web (reminder not to delete)
9. Guide participant to create i-ABC account through the link sent previously via email and complete demographics questionnaire.

Steps for setting up Inquisit Tasks (Web version) (for participants):

*Note: All tasks have been tailored specifically to run on Inquisit 5 and all participants are required to use ONLY Inquisit 5 to run the above tasks.

1. Download Inquisit 5 Web: depend on your operating system
    a. https://www.millisecond.com/download/inquisitweb5.aspx
    b. Ask participant to check using another link https://mili2nd.co/ilsb
2. Click on the green button "Run" to start the experiment

After Session 1: Checklist (for experimenters)

1. Obtain help for trouble-shooting if there were any unresolved hardware/software issues.
2. Purchase the appropriate E-voucher selected by the participant.

Between Session 1 and Session 2:

1. Send a reminder email one day before Session 2.

Session 2 Checklist (for experimenters)

1. If the experimenter is different from Session 1, please ensure that you obtain the correct Testing Environment Checklist for the respective participant and complete the Session 2 column.
2. Ensure that audio/sound playback is at comfortable level (Play first 15 seconds of the audio clip).
3. Remind participant to adjust screen brightness to maximum.
4. Check that participant is using a computer mouse and that it is in working condition.
5. Send participant the link for each task in the Teams chat when appropriate.
6. Check that participant is using the MS Teams desktop app is used and request participant to share their screen through the desktop (not window) while completing tasks (to ensure that all tasks can be seen by the experimenter at all times).

7. Ask participant to turn off all running background apps and mute desktop notifications to avoid distractions during task completion.
8. Ensure that participant's web browser is maximised fully before attempting tasks.
9. Experimenter to disable their own video function before start of each task and to remind participant to minimize the video screen panel.
10. Conduct experimental tasks:
    a. Check the Task order.
    b. Download the correct Task Checklist according to Task order no. for exact sequence of tasks.
    c. Remind participant to inform experimenter if they leave for a short break in between blocks of a task (e.g., Structure Learning).
    d. Ask participant if they need to take a short break before starting long duration tasks e.g., Structure Learning
    e. For iABC tasks, ask participants to click on the "Done" button after each task so that the data is properly saved.
    f. For the Backwards Digit Span task, experimenter to share screen with system audio via Teams and play audio recordings aloud - volume adjusted to maximum.
