# Supplementary material for: A New Remote Guided Method for Supervised Web-Based Cognitive Testing to Ensure High-Quality Data: Development and Usability Study
Source: J Med Internet Res. 2022 Jan 6;24(1):e28368. doi: 10.2196/28368 (PMC8778570; doi:10.2196/28368)
Supplement: Multimedia Appendix 2 [file jmir_v24i1e28368_app2.pdf]

Multimedia Appendix 1: Equipment Questionnaire (RGT)

Q1 Participant ID : ____

Q2 My computer is a...

- ◯ Laptop
- ◯ Desktop
- ◯ Other ____

Q3 The brand of my computer is:

- ◯ Apple
- ◯ HP
- ◯ Lenovo
- ◯ Dell
- ◯ Acer
- ◯ Fujitsu
- ◯ Asus
- ◯ Other ____

Q4 The processor in my computer is:

- ◯ Intel Core i3
- ◯ Intel Core i5
- ◯ Intel Core i7
- ◯ Intel Core i9
- ◯ Other ____

Q5 My computer RAM is (note minimum is 8GB):

- ◯ 8GB
- ◯ 16GB
- ◯ 32GB
- ◯ Other ____

Q6 My computer's free hard disk space is:

- ◯ 256GB
- ◯ 512GB
- ◯ 1TB
- ◯ Other ____

Q7 My computer's total hard disk space is:

- ◯ 256GB
- ◯ 512GB
- ◯ 1TB
- ◯ Other ____

Q8 My physical display screen size in inches, measured diagonally is:
(Please only state the numerical value of the screen size, eg. 13)

Q9 My computer's screen refresh rate is (in Hertz):
(Please only state the numerical value of the refresh rate, e.g., 60)

Q10 My computer's screen resolution is (in pixels):
(Please only state the numerical value of your screen resolution, e.g., 2560 x1600)

Q11 My mouse is (Note that you need a mouse for the testing session):

- ◯ Wired
- ◯ Wireless

Q12 My keyboard is:

- ◯ Wired
- ◯ Wireless
- ◯ Integrated
- ◯ Other ____

Q13 My webcam model is:

- ◯ Integrated
- ◯ Separate Device (Write the model) ________________________
- ◯ Other ____

Q14 My microphone is:

- ◯ Integrated
- ◯ Separate Device (Write the model) ________________________
- ◯ Other ____

Q15 My operating system and the version operating system:
eg. Mac OSX Mojave Version 10.14.6

____

Q16 My web browser is:

- ◯ Google Chrome (Recommended)
- ◯ Safari
- ◯ Internet Explorer/ Edge
- ◯ Mozilla Firefox
- ◯ Other ____

Q17 Please select the range of your download speed:
You can check this at this link: https://speed.measurementlab.net/#/

- ◯ 0-5 Mb/s
- ◯ 6-10 Mb/s
- ◯ Other ____

Q18 Please select the range of your upload speed:
You can check this at this link: https://speed.measurementlab.net/#/

- ◯ 0-5 Mb/s
- ◯ 6-10 Mb/s
- ◯ Other ____

Q19 Please select the range of your latency speed:You can check this at this link: https://speed.measurementlab.net/#/

- ◯ 0-5 ms
- ◯ 6-10 ms
- ◯ Other ____

Q20 Any additional details that you would like to add about your hardware device:

____
____
____
