# Supplementary material for: A New Remote Guided Method for Supervised Web-Based Cognitive Testing to Ensure High-Quality Data: Development and Usability Study
Source: J Med Internet Res. 2022 Jan 6;24(1):e28368. doi: 10.2196/28368 (PMC8778570; doi:10.2196/28368)
Supplement: Multimedia Appendix 3 [file jmir_v24i1e28368_app3.pdf]

Multimedia Appendix 2: Testing Environment Checklist (RGT)

Participant ID: ______________

**Testing Environment Variable**	**YES (1) / NO (0) Note 1 or 0**		**Comments**
	**Session 1**	**Session 2**	
Quiet Location			
Room with door closed			
Bright lighting			
Minimal Background noise			
Any potentially sensitive information in background			
If applicable, is laptop plugged in and charged			
Handphone in silent mode or switched off			
Handphone placed faced down			
Any other Disruption/interruption			

Checked by: _________________
