# Supplementary material for: A New Remote Guided Method for Supervised Web-Based Cognitive Testing to Ensure High-Quality Data: Development and Usability Study
Source: J Med Internet Res. 2022 Jan 6;24(1):e28368. doi: 10.2196/28368 (PMC8778570; doi:10.2196/28368)
Supplement: Multimedia Appendix 4 [file jmir_v24i1e28368_app4.pdf]

Multimedia Appendix 3: Task Descriptions & Performance Indices

1. Wisconsin Card Sort Test (WCST)

The WCST is a measure of cognitive flexibility that examines rule acquisition and set shifting [13-14]. Participants were presented with four stimulus cards and a deck of response cards that varied on three dimensions (color, shape, and number of geometric figures, see Figure C1a). They were asked to match a fifth card from the sequentially presented response cards to one of the four stimulus cards as accurately as possible within 5 seconds. There were various potential rules that could underpin the classification, for instance matching the cards by shape, number, or color. Participants were required to identify and apply the correct card classification rule in accordance with the feedback they received after each trial. This feedback was presented visually through the appearance of either a tick or a cross on the screen (see Appendix C1b). After 10 trials, the matching rule changed, and participants were required to find the new rule and adjust their response accordingly. The total number of trials was 60 and the task lasted approximately 5 minutes.

This version of the WCST was based on the original formulation by Berg (1948 [15]), which included 4 stimulus and 60 response cards. The response deck therefore contained all possible permutations of 4 possible colours, 4 possible shapes and 4 possible numbers (total of 64) *except for* the exact combination of features shown in the 4 stimulus cards. It should also be noted that although computerised versions of the WCST are now widely used [16-17] and have demonstrated comparable split-half reliability on error measures to paper-based versions of the task [18], the current iABC version has not yet been formally validated and is therefore experimental.

Figure C1.(a, top) The four types of shapes that constitute each WCST card stimuli. (b, bottom) WCST stimuli presentation and feedback screens. For clarity, red boxes indicate the participant's selected responses, but were not actually shown on screen.

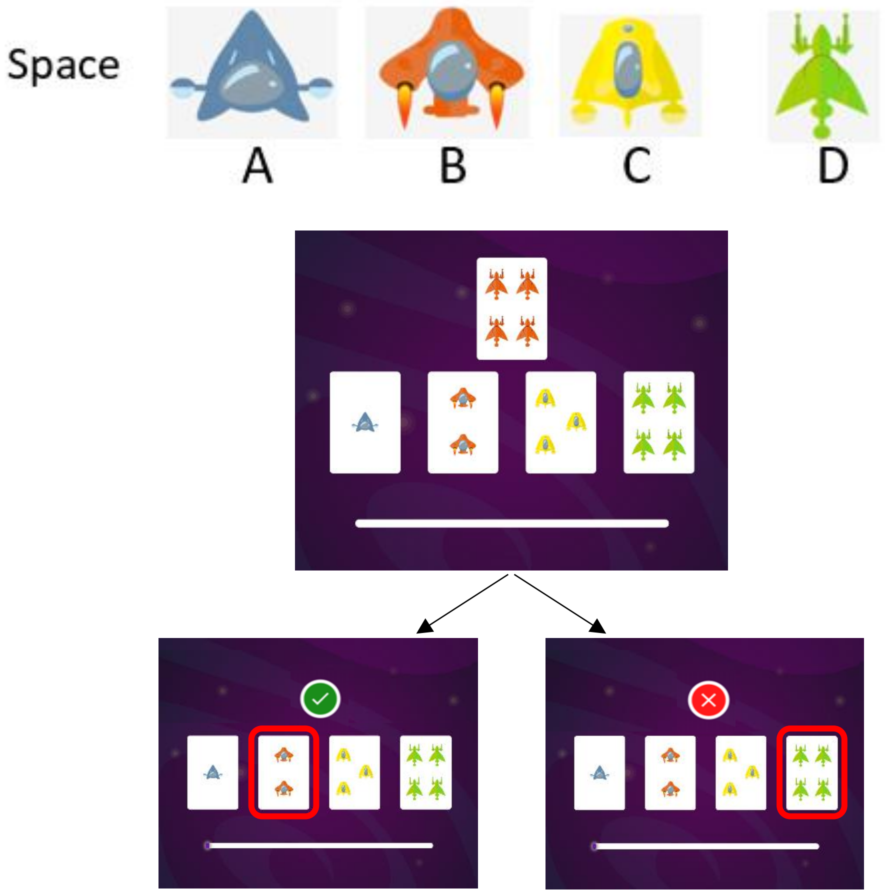

Performance on the WCST was assessed by analysing the number of trials the participant took to correctly identify a new rule after it had changed. Two key indices were computed: perseverative and non-perseverative errors. If participants persisted with the previous rule after a change in rule, the number of errors in this pattern were considered as perseverative errors. Any error which was not a perseverative error was considered as a non-perseverative error. These two indices were selected as perseverative errors are a sensitive index of cognitive flexibility with obvious face validity. Non-perseverative errors reflect a more general measure of performance/learning which can be used as a control for the specificity of the cognitive flexibility effect. Those subjects showing selective increases in perseverative errors therefore may be said to exhibit reduced cognitive flexibility.

2. Probabilistic Learning and Reversal (PR) Task

In the PR task, participants were presented with two different coloured visual icons and instructed to select one of them within 5 seconds. The stimuli used in this test were pairs of blue- and yellow-coloured rockets which might appear in any two out of four locations (boxes) on the screen (Figure C2). The instructions reminded participants to choose the pattern that was most frequently correct and notified them that the rule which defined the correct pattern might change throughout this task though this would not happen often. In the first discrimination phase, the participant selected one of the two icons and was provided feedback about whether their response was correct or incorrect. Response feedback was probabilistically determined (80-20) with one icon yielding positive feedback 80% of the time (and negative feedback 20%), whereas the other only yielded positive feedback 20% of the time (and negative feedback 80%). In the second reversal phase, these probabilities were reversed. Each phase consisted of 40 trials, giving a total of 80 trials. The task duration was approximately five minutes.

Two indices were used to assess performance on the PR task: (a) Perseveration. In the reversal phase, the rule was reversed and the number of trials that the participant required to update their response in accordance with the new rule after reversal was computed as the perseveration index. (b) Switching Probability. This was the total number of times that the participant changed their response when negative feedback was displayed.

Figure C2. PR task stimuli presentation and feedback screens. For clarity, red boxes indicate the participant's selected responses, but were not actually shown on screen.

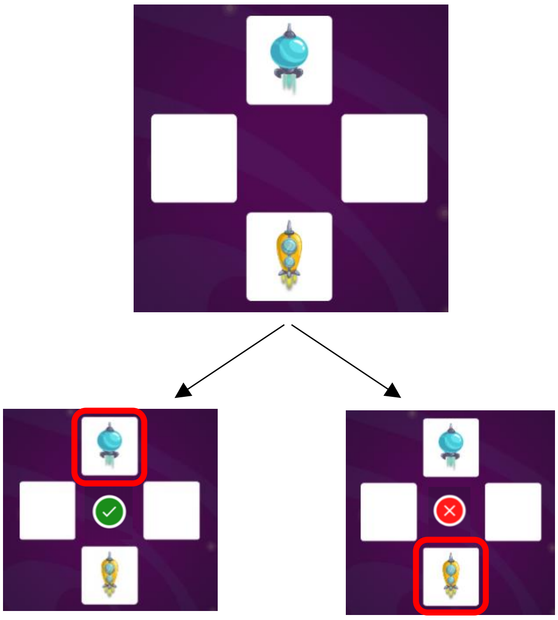

3. Structure Learning (SL) Task

This task involved making predictions based on previously learned patterns in a series of visual stimuli. The probabilistic sequences were determined by Markov models. The SL task was produced by Cambridge University's Adaptive Brain Lab. Here, participants would see a sequence of visual symbols [19] before they were required to predict which symbol should come next in the sequence. The symbols consisted of four sets: A, B, C or D (Figure C3a). These symbols were selected from Ndjuká syllabary and were highly distinct from one another [19]. Each symbol was presented in black on a grey background. The default stimuli size was 225 pixels and the stimulus size was re-scaled following the screen size of each participant to ensure a consistent screen-to-stimulus size ratio across all participants.

Figure C3a. Sets of symbols for SL task stimuli

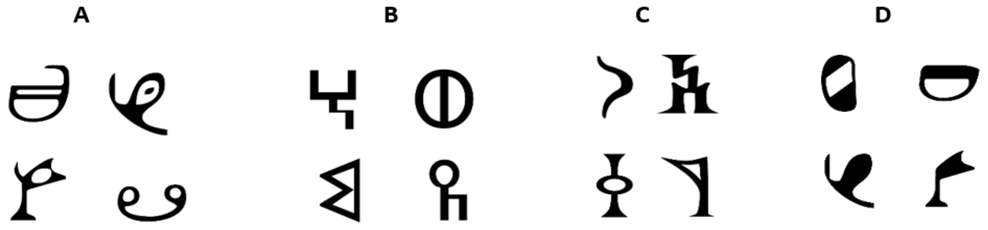

The task had multiple levels. Level-0 was the simple probabilistic presentation of symbols i.e. out of the 4 symbols, one appeared with a high probability, one with a low probability and the other two rarely appeared. For example, the probabilities of occurrence for the four symbols A, B, C, and D were 0.18, 0.72, 0.05, and 0.05, respectively. Presentation of a given symbol was independent of the preceding stimuli. At Level-1 the target stimulus depends on the stimulus immediately preceding it. That is, given a preceding symbol, only two targets could appear, one with a high probability (e.g., 80%) and the other with a low probability (e.g. 20%). For example, when symbol A was presented, only symbols B or C were allowed to follow, and B had a higher probability of occurrence than C.

There were three task phases: a mandatory practice phase, followed by an optional practice phase and lastly the test phase. The mandatory practice phase had to be completed before a test phase could proceed and was only presented once per participant. If the participant chose to repeat this phase, the optional practice phase was launched; if not, participants continued to the test phase. Each practice phase had one block consisting of 5 trials, presenting symbols from set D in a random order. The test phase consisted of 7 blocks of 60 trials each, presenting symbols from sets A, B or C (randomly assigned by participant).

During the task presentation, each trial varied in length between 9-13 symbols. During each trial, every symbol was shown for 100ms, followed by a white dot fixation point appearing mid-screen for 400ms. The trial concluded with a response screen showing four symbols in a 2 x 2 grid. Participants indicated which of these might appear next in the sequence by clicking on one of the four symbols, after which they moved on to the next trial. If there was no selection made, the response screen would time-out after 2000 ms and the next trial would begin (Figure C3b). To prevent participants from learning the spatial location of the target response stimuli, the positions of the symbols were randomised. At the end of each block, participants received feedback on their performance. The task duration was approximately 50 minutes.

Figure C3b. SL task example trial sequence

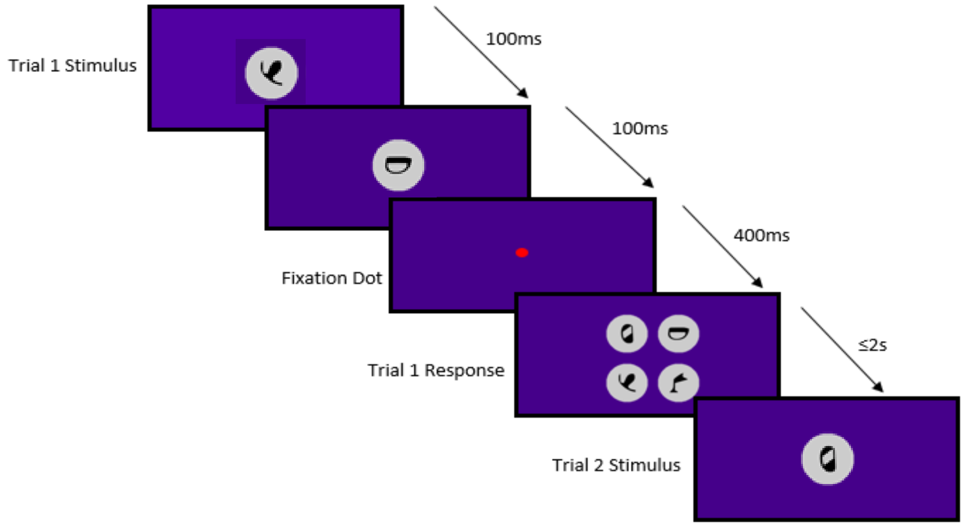

In this task, participants were required to predict the next symbol to appear in a sequence of symbols shown. Even though the order of the symbols appeared stochastic, this was based on a Markov model which controlled the transitional probabilities between symbols. Structure learning is highly probabilistic in nature and hence responses cannot be analysed solely on the basis of accuracy. Following previous studies, responses were analysed in a probabilistic manner [19]. The absolute Euclidean distance (AbDist) between the distribution of participant responses and the distribution of presented targets was estimated across the 60 trials for each block as shown in Eq(1).

$$AbDist(context) = \Sigma\, |Pr(target|context) - Pp(target|context)|$$

Eq(1)

AbDist was computed for each context as the sum over all targets in the symbol set of | Pr() – Pp() |, where Pr() denotes the probability distribution of participant responses to each target symbol, and Pp() denotes the probability distribution of the presented symbols. The minimum overlap between the two distributions was then quantified by calculating a Performance Index (PI) for each context, as follows:

$$PI(context) = \Sigma\, min(Pr(target|context), Pp(target|context))$$

Eq(2)

Hence, PI(context) = 1 - AbDist(context)/2. This measure was then averaged across contexts and weighted by the corresponding stationary context probabilities as follows:

$$PI = \Sigma\, PI(context)\,.P(context)$$

Eq(3)

Finally, the Performance Index was normalised by subtracting the expected Performance Index for random guessing (i.e. equal probability for each target). This normalised PI was computed for each experimental block, and the mean PI across all blocks was used as the first outcome measure. The second outcome measure, PI change, was defined as the difference in performance between the first two blocks (blocks 1 and 2) and the last two blocks (blocks 6 and 7) of the experiment.

4. Stroop Task

The Stroop colour-word task is a well-known measure of cognitive inhibition [20]. In each trial, the participant is presented with a colour word (e.g. “RED” printed in green colour) shown in the centre of the display screen. They are required to respond by indicating the physical colour of the text while ignoring the word itself (Figure C4). Four colours were used (red, green, blue, yellow) and these were mapped to four corresponding response keys on the keyboard (d,f,j,k for each colour respectively). This response button mapping was displayed at the top of the screen for all 180 trials to aid participants and reduce their memory load. Participants were instructed to respond as quickly and accurately as possible, with their response being timed. Once a response was made, the next trial followed. If an incorrect response was made, a red cross flashed on the screen. Before proceeding with the first trial, the participant was reminded to place their index and middle fingers on the ‘d’, ‘f’, ‘j’, ‘k’ keys. On congruent trials (n = 144), the print colour and the word meaning matched (e.g., “RED” printed in red). On incongruent trials (n = 36), the print colour and word meaning were mismatched (e.g., “RED” printed in green).

For each trial, the reaction time (RT) in milliseconds was recorded from the start of the word stimulus presentation to the participant’s response. The reaction times along with the accuracy for both congruent and incongruent trials were measured. For analysis of interference effect, the reaction times of only the correct responses were considered. Two interference indices were calculated based on the difference in reaction time and accuracy respectively between the congruent and incongruent stimuli.

Figure C4. Example of Stroop Task presentation screen

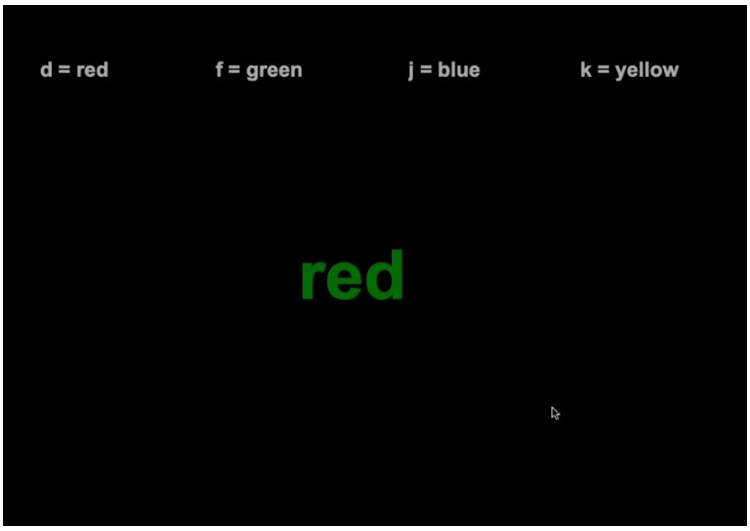

5. Stop Signal Task (SST)

The Stop Signal Task is a measure of response inhibition and impulse control [21,22]. Prior to beginning the task, participants were shown an instructions screen and completed practice trials. In each trial, participants saw an arrow in the middle of the screen that either pointed to the right or left (Figure C5). Participants had to press the left response key “d” if the arrow pointed to the left and the right response key “k” if the arrow pointed to the right, responding as quickly as possible. On certain trials, an auditory signal (a brief tone) was played after the presentation of the arrow. On these “stop” trials, participants were required to refrain from executing their response. The time delay between the presentation of the arrow and the signal tone was adjusted adaptively based on the participant’s performance, starting at 250 ms and decreasing or increasing in steps of 50 ms. The delay was increased if

the previous stop trial was successful (up to 1150 ms) or decreased if the previous stop trials was not successful (down to 50ms). Each trial lasted for 2000 ms and participants could respond at any point during this period. There were four blocks in total: one practice block with 32 trials (8 stop signal trials, 24 no signal trials) and three test blocks of 64 trials each (16 stop signal trials, 48 no signal trials). The duration of this task was 12 minutes.

Figure C5. SST Task stimulus presentation screen for right- and left-pointing arrows

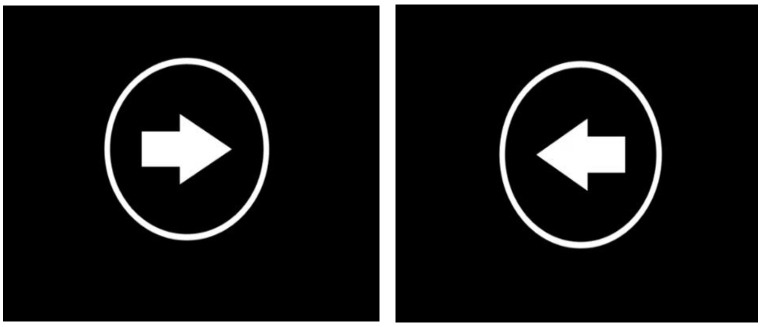

Performance on this task is dependent on the relative finishing time of two processes [22]: a go process, triggered by the presentation of the primary-task stimulus, and a stop process, triggered by the presentation of the stop signal. If the stop process finishes before the go process, participants successfully inhibit their response. If the go process finishes before the stop process, response inhibition fails, and participants respond when they should not. The latency of the stop process, or Stop Signal Reaction Time (SSRT), was estimated using the integration method following Verbruggen et al [22]. Specifically, the integration method involves "integrating" the reaction time (RT) distribution and identifying the nth RT, where n is the total number of response times in the RT distribution of go trials multiplied by the participant's probability of responding on a stop-signal trial (i.e., p(respond|signal)). The SSRT is then calculated by subtracting the mean Stop Signal Delay (SSD) from this identified RT value in the nth RT position. This version of the integration method is thought to produce the most reliable and least biased non-parametric SSRT estimates.

6. Trail Making Test

The Trail Making Test [23] is a neuropsychological test of visual search speed and motor tracking ability. It is also implicated in cognitive flexibility and in the ability to maintain sets [24]. The computerised version used here was based on the Comprehensive Trail-Making Test (CTMT, [25]) which features empty distractor circles in addition to the classic Trails forms (see Figure C6a). The Trail Making Test consists of two sub-tests: Part A and Part B. In Part A, the participant was presented with a visual display containing 25 numbered circles positioned in a random order. They were instructed to use their computer mouse to draw a continuous line connecting the circles in sequential order (e.g., 1-2-3…) as quickly as possible (see Figure C6b). Participants were told to hold down the mouse button while drawing. If the mouse was accidentally released from the participant's grip, a yellow circle would appear, highlighting where they last stopped. In Part B, the stimuli consisted of a mixture of numbers and letters. Participants had to connect the circles in an alternating order between numbers and letters (i.e., 1→A→2→B). A total of 13 circles containing numbers 1-13 and 12 circles containing letters A-L were presented (Appendix C6b). In addition, there were 15 empty distractor circles.

Two reaction times were recorded for the Trails A and Trails B tasks respectively. The Trails A reaction time was the total time taken by the participant to complete connecting

all numbered circles in the correct sequential order. Similarly, the Trails B reaction time was the time taken by the participant to complete connecting circles, alternating between letters and numbers in the correct order. To estimate performance, the ratio of the Trails B to Trails A reaction time was computed.

Figure C6. (a, left) Trial Making Test Part A with 25 numbered circles. (b, right) Trail Making Test Part B with 25 circles labelled with numbers and letters

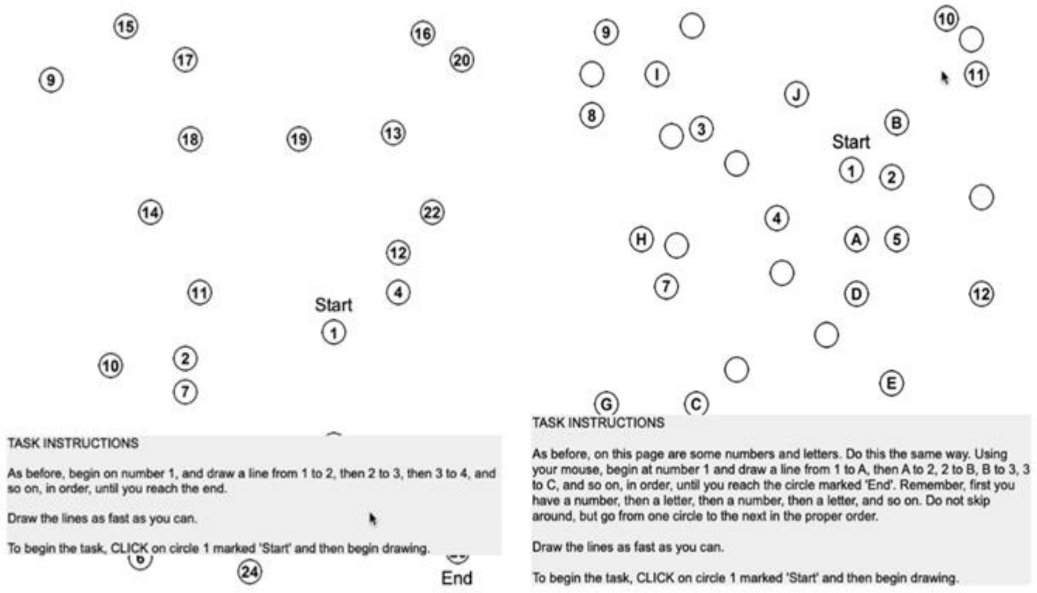

7. Intra-Extra Dimensional Set Shift task (IED)

The IED task is a test of rule acquisition and reversal. It requires visual discrimination, attentional set formation and maintenance, shifting and flexibility of attention [26]. Participants advance through nine sequential task stages. On each trial, participants are presented with a pair of stimuli and they are required to select the correct stimulus based on an underlying rule which must be figured out and learned by trial and error. In the first two stages, the stimuli consist of simple white lines only (Figure C7a). From stage 3 onward, the stimuli increase in complexity and consist of purple shapes and white lines which are overlaid (Figure C7b). Participants respond by clicking on one of the two stimuli and then receive feedback about whether their selection was correct. After six consecutive correct responses, the rule and/or the stimuli change, though this transition is not made known to the participant. The new rule is either based on the same feature of the stimuli i.e., white lines that guided the selection rule in the prior stage (intra-dimensional shift) or on the other stimulus feature i.e., purple shape (extra-dimensional shift). The test duration was 7 minutes.

Figure C7. (a, left) IED Task Stage 1 and 2 stimuli. (b, right) IED Task stimuli used from Stage 3 onwards

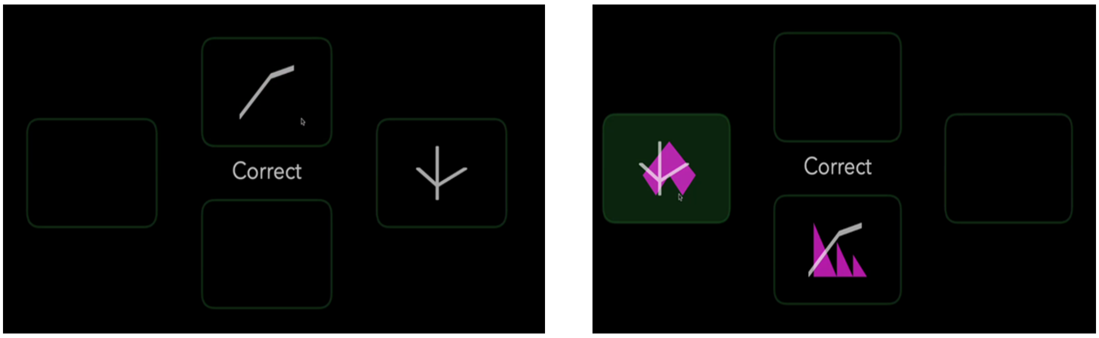

The IED is a nine-stage task, and the rule for the correct response was modified at the start of each stage. Specifically, intradimensional rule shifts (IDs) assessed participants' ability to generalize a rule to new stimuli whereas extradimensional shifts (EDs) assessed cognitive flexibility as the ability to shift attention to a previously irrelevant dimension [66]. The two key performance indices in this task were the number of errors made to complete the stage where there was an ED shift (Stage 8), and the total number of errors made in the stages prior to the ED shift.

8. Spatial Working Memory (SWM) Task

The SWM task measures memory retention and manipulation of visuospatial information [27]. The task begins with a set of coloured squares (boxes) shown on the screen. The participant is required to search the boxes for a yellow 'token' (which then fills up an empty column on the right-hand side of the screen). Once found, a second yellow token is then hidden behind one of the remaining boxes, although all the original boxes remain available on screen. Participants have to remember which boxes have previously been successfully searched and narrow their search by a process of elimination. There are 5 stages with increasing levels of difficulty (3, 4, 6, 8 and 12 boxes/tokens respectively). An example is shown in Figure C8. The colour and position of the boxes used are changed from trial to trial to discourage the use of stereotyped search strategies. The test duration was 4 minutes.

Figure C8. SWM Task illustrating three tokens

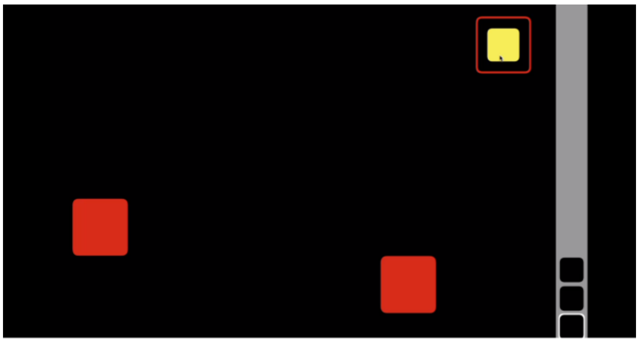

The two key measures for this task related to errors and strategy. SWM Between Errors were computed as the number of times that the participant incorrectly revisited a box, calculated across 4, 6, 8 and 12 token trials. SWM Strategy which was the number of unique boxes from which a participant started a new search in the 6 and 8 box trials. More efficient searches are carried out by searching boxes in a fixed order [27].

9. WASI-II Vocabulary Test

To assess verbal intelligence, the Vocabulary subtest of the Wechsler Abbreviated Scale of Intelligence - Second edition (WASI-II; [28]) was administered. Participants were asked to provide a verbal definition of an English word such as "haste". A total of 22 word items were presented with increasing complexity. The experimenter prompted or sought clarification from the participant if their answer was unclear, too vague to be readily scored, or specifically necessitated a prompt as defined in the administration manual. The experimenter prompted the participant by asking "What do you mean?" or "Tell me more about it". Each participant was video or voice recorded for scoring and review purposes. The test usually took between 5 to 10 minutes to complete.

In the 22 word-item list, each item was scored as either zero, one or two depending on the detail and accuracy of the answer given. The raw score out of 59 was taken as the

performance index, with 0 being the lowest and 59 being the highest score possible. This raw score was then transformed into standardised T-scores by participant age.

10. Backward Digit Span (BDS) Test

The BDS subtest from the Weschler Adult Intelligence Scale- Fourth Edition (WAIS-IV; [29]) is a test of verbal working memory. Participants were told to listen to an audio clip which presented a sequence of digits of increasing length (2 to 9 digits). Each clip was only played once. After listening to each audio clip, participants had to recall aloud each sequence in reverse order. Each trial consisted of two sequences of the same length. If participants gave a correct response to at least one of the two sequences for a specific digit length, the experimenter would proceed to the next length. When the participants gave incorrect responses to both items of equal length, the test would end. The score given for a correct response was 1 and for an incorrect response was 0. Out of the 2 sequences per length, the participant had to obtain a minimum score of 1 to proceed to the next sequence. The raw score out of 16 was taken as the performance index, with 0 being the lowest and 16 being the highest score possible. A total of 16 trials were presented. This test required about 5 to 10 minutes to complete.

The backward digit span task is more complex and cognitively demanding than the forward span task as it necessitates transformations in working memory in addition to simple storing of information and attentional processes [30,31]. We used only the backward span task as this more difficult task better captured variations in performance in our healthy young adult participants, permitting more sensitive detection of test modality differences, if any.
